# Transition to dietary plant-based proteins requires functional liver GDH for proper control of ammonia and energy homeostasis

**DOI:** 10.1016/j.molmet.2026.102435

**Published:** 2026-09-04

**Authors:** Yan Zhou, Angela M. Ramos-Lobo, Tingting Fu, Hector Gallart-Ayala, Julijana Ivanisevic, Pierre Maechler

**Affiliations:** 1 Department of Cell Physiology and Metabolism, University of Geneva Medical School, Geneva, Switzerland; 2 Department of Quantum Matter Physics, Laboratory of Advanced Technology, University of Geneva, Geneva, Switzerland; 3 Metabolomics Platform, University of Lausanne, Lausanne, Switzerland

**Keywords:** Liver, Dietary proteins, Gluconeogenesis, Glutamate dehydrogenase

## Abstract

Plant-based diet is growingly recommended, although switching the source of proteins from animal to plant regimen requires metabolic adaptations. According to its pivotal function in bridging amino acids and glucose homeostasis, we investigated the role of liver GDH in short-term adaptation to plant versus animal dietary proteins keeping the same spectrum of macronutrients. Control and Hep-*Glud1*^*−/−*^ mice lacking liver GDH were fed for 4 days with either animal- or plant-based diets. Hepatic GDH was upregulated in control mice eating animal proteins compared to mice under the plant regimen. Glycemia of ad libitum fed control mice were lower under plant-based regimen compared to animal-based diet; not further changed in Hep-*Glud1*^*−/−*^ mice. After 6 h of fasting, glycemia remained within the physiological range but mice under the plant-based diet engaged a more robust fasting response versus animal protein regimen. In Hep-*Glud1*^*−/−*^ mice, exhibiting impaired endogenous glucose production evoked by amino acids, plant-based diet was associated with reduced plasma concentrations of gluconeogenic alanine and L-carnitine precursor lysine, underscoring limited fasting adaptation. Liver *in situ* high-resolution metabolomics revealed higher pyruvate levels in periportal regions of mice fed the animal-based diet and the knockout of liver GDH induced elevated aspartate, while urea production was impaired. This resulted in high plasma ammonia concentrations in Hep-*Glud1*^*−/−*^ mice, causing severe hyperammonemia in GDH null mice under plant-based diet. These mice exhibited reduced voluntary physical activity, an effect absent in Hep-*Glud1*^*−/−*^ mice under animal-based proteins. Overall, dietary source of proteins shapes energy partitioning according to the respective amino acid availability.

## Highlights

• Plant-based diet is recommended but little is known about metabolic adaptations.
• We report the role of liver GDH in short-term response to plant versus animal diet.
• A diet based on animal proteins confers more robust glycemic control.
• Lacking liver GDH results in severe hyperammonemia upon plant-based food.
• Liver GDH is central for maintenance of ammonia homeostasis and physical activity.

## 1 Introduction

In recent years, plant-based food has been progressively more recommended for reasons related to environmental impacts, animal welfare, and human health [1]. Current recommendations state that a healthy diet with appropriate caloric intake should consist of a diversity of plant-based foods and low amounts of animal sources [2]. Within the range of animal-based foods, red meat consumption has been associated with a greater risk of cancer [3], cardiovascular diseases and type 2 diabetes [4]. However, compared to animal-derived proteins, exclusive consumption of plant proteins has also been associated with lower muscle mass [5]. Reducing meat consumption while keeping an animal source of proteins reduces the risk of type 2 diabetes, as reported in pesco- and semi-vegetarians benefiting from intermediate protection, that is further improved by lacto-ovo vegetarian diet, compared to nonvegetarian diet [6].

A possible explanation for the relative benefit of animal-based proteins could be that proteins from plant sources have lower digestibility and provide less essential branched-chain amino acids, which may participate in the preservation of the muscle mass on the one hand but, on the other, have also been associated with insulin resistance [7,8]. Among those amino acids, leucine is an allosteric activator of glutamate dehydrogenase (GDH), a mitochondrial enzyme playing a key role in both hepatic ammonia detoxification and gluconeogenesis [9]. Through deamination of glutamate, GDH provides α-ketoglutarate to the tricarboxylic acid cycle, thereby ensuring anaplerotic route required for gluconeogenesis, as well as free ammonia NH_3_ used by the ornithine cycle for the production of urea and safe disposal of the potentially toxic ammonium cation NH_4_^+^ [10,11]. This illustrates the pivotal role of liver GDH that must accommodate various physiological conditions with continuous changes in the provision of amino acids according to diets and postprandial versus fasting states.

Upon fasting, gluconeogenic pathways gradually take over glycogenolysis to maintain adequate endogenous glucose production. This requires the release of gluconeogenic amino acids from the skeletal muscles, predominantly glutamine and alanine. After being deaminated in the liver, their respective products provide substrates for *de novo* glucose production. This balance of substrate availability is shaped by food composition, with an important contribution of the profile of amino acids according to the source of dietary proteins. Studies dedicated to the impact of plant versus animal diets are typically based on populations or groups of people routinely used to their respective diets [5,12]. Little is known about short-term adaptive responses when switching from one to the other diet in the same individuals. It was reported that, compared to a diet entirely based on plants, consumption of animal-based diet for five consecutive days is sufficient to change the human gut microbiome [13]. Such rapid adaptation of the gut microbiome to food composition mirrors differences observed between carnivorous and herbivorous mammals [14]. Regarding the liver, its rapid metabolic adaptation to the fasting state is well established [15]. However, we have a poor understanding of hepatic changes upon qualitative variations in food composition, such as keeping the same spectrum of macronutrients while changing the source of those. Switching from animal to plant proteins or vice et versa alters the profile of amino acids made available for the organism, with the liver as a major hub of their final catabolism, and hepatic GDH playing a central role in this process.

According to its pivotal function in bridging amino acids and glucose homeostasis, we investigated the role of liver GDH in the short-term adaptative response to plant versus animal dietary proteins. Specifically, the source of proteins for the animal-based diet was milk-derived casein and the plant food was made of various cereals. Accordingly, the animal-based diet can be referred to as lacto-vegetarian and the plant-based one as vegan diet [12], although the former is essentially composed of animal proteins. Thus, we compared adaptation to food having the same 20% protein contents, whereas in the plant-based diet the proportion of essential branched-chain amino acids was lower, while tryptophan content was higher, compared to animal-based diet. The results show that, compared to plant-based proteins, a diet based on animal proteins conferred a more robust glycemic control relying on hepatic GDH in the fasting state. Lacking liver GDH resulted in marked hyperammonemia when fed the plant-based food and negatively impacted on spontaneous physical activity.

## 2 Methods

### 2.1 Animals and diets

Deletion of GDH in hepatocytes was induced *in vivo* by tamoxifen treatment of *Glud1*^*lox/lox*^ male mice (*Glud1*^*tm1.1Pma*^, MGI:3835667 [16]) carrying the Albumin*-CreER*^*T2*^ construct [17] generating liver-specific GDH-knockout (Hep-*Glud1*^*−/−*^) mice characterized previously [9]. The recombination was induced at 8 weeks of age by subcutaneous implantation of tamoxifen pellets (Tamoxifen free base, 25 mg/pellet, 21-day release, E−361; Innovative Research of America). Experiments were conducted 4 weeks later when mice reached 12 weeks of age. Implantation was also performed in control floxed *Glud1*^*lox/lox*^ (Control-*lox*) mice composed of littermates to optimize standardization of the genetic background between the two groups. Mice were maintained in our certified animal facility (12 h × 12 h light/dark cycle with 7:00 am on and 7:00 pm off) according to procedures approved by the animal care and experimentation authorities of the Canton of Geneva (GE/166/16). Food and water were provided ad libitum during the duration of the experiment unless otherwise specified.

At 12 weeks of age, the mice were divided into two groups and fed for 4 days with diets with 20% (w/w) proteins from either animal (casein; D12450B, Research Diets, New Brunswick, NJ) or plant (cereals plus brewer's yeast; SAFE 150, Safe, Rosenberg, Germany) sources; see Supplementary Table S1 for overall composition. Sacrifice and tissue collection were conducted under both ad libitum fed state (food available) and after a physiological [18,19] 6 h fasting period (food removal: 8:00 am to 2:00 pm). Prior to sacrifice, glycemia was measured using an Accu-Check Aviva glucometer (Roche Diagnostics). At sacrifice, blood samples were collected in sodium-heparin tubes and derived plasma as well as tissues of interest were stored at −80 °C for further analyses.

### 2.2 In vivo experiments and metabolic parameters

Metabolic phenotyping was assessed using a TSE PhenoMaster system (TSE Systems, Berlin, Germany) for monitoring of calorimetric parameters (O_2_ consumption and CO_2_ production), food and water intake, as well as spontaneous locomotor activity and voluntary running wheel activity.

Glutamine and β-hydroxybutyrate were determined on plasma kept at −80 °C up to 2 weeks using commercial kits (ab197011 from Abcam and K632-100 from Biovision, respectively). ELISA kits used to assess plasma levels of insulin and glucagon were from Crystal Chem (Elk Grove Village, IL) and from Enzo (Farmingdale, NY) for cortisol. Plasma ammonia and urea were measured within 20 min after blood collection using a COBAS C111 analyzer (Roche Diagnostics).

Endogenous glucose production was evaluated by an amino acid challenge following 6 h of fasting and i. p. injection of 1 g/kg L-alanine and 1 g/kg L-glutamine (Sigma–Aldrich, Burlington, MA). After amino acid administration, blood samples were taken at the indicated time intervals (0–120 min) for glucose measurements using Accu-Check Aviva glucometer. The total area under the curve (AUC) of glycemia was calculated using the GraphPad Prism 10 software.

### 2.3 Immunodetection on western blotting and immunohistochemistry

For western blotting, protein extracts (30 μg) were prepared from tissues homogenized in RIPA buffer and separated on bis-acrylamide 10% gel, then probed with specific antibodies (see detailed list, suppliers, and dilutions in Supplementary Table S2). Bands were revealed by horseradish peroxidase assay and quantification was performed using the Syngene PXi gel imaging system (Thermo-Fischer).

For immunohistochemistry, liver cryosections were fixed in cold acetone and then blocked with normal goat serum before overnight incubation at 4 °C with primary antibodies against GDH or GS (see suppliers and dilutions as in Supplementary Table S2). On the next day, sections were washed with PBS and incubated with secondary antibodies at room temperature for 2 h; then washed again in PBS before image acquisition on Leica LSM800 Airyscan microscope (Leica, Wetzlar, Germany).

### 2.4 Metabolomics

LC-MS metabolic profiling of liver and plasma samples was conducted to determine concentrations of amino acids and gluconeogenic intermediates. In brief, plasma and liver amino acids were analyzed by Hydrophilic Interaction Liquid Chromatography coupled to tandem mass spectrometry (HILIC - MS/MS) in positive ionization mode using a 6495 triple quadrupole system (QqQ) interfaced with 1290 UHPLC system (Agilent Technologies), as previously described [20]. Targeted quantification of intermediates in glycolysis and TCA cycle was analyzed in liver samples by HILIC - MS/MS in negative ionization mode using a 6495 triple quadrupole system interfaced with 1290 UHPLC system, as reported [21].

*In situ* metabolomics was performed on liver cryosections using high resolution time-of-flight secondary ion MS (ToF-SIMS) imaging to allow metabolite mapping at the cellular scale [22]. For sample preparation, the frozen tissues embedded in OCT medium were sectioned at 8 μm thickness in a cryostat. Tissue sections were mounted onto stainless-steel slides and immediately transferred to −80 °C deep freezer. Prior to the ToF-SIMS imaging, the cryosections were brought to room temperature and dried in a desiccator under low vacuum for 30 min. To spatially navigate within the sections, ToF-SIMS was combined with GS immunostaining performed on subsequent sections to identify pericentral (GS-positive) versus periportal (GS-negative) regions of interest (ROI). ToF-SIMS imaging was performed using a PHI nanoTOF II ToF-SIMS instrument (Physical Electronics) equipped with a 30 kV bismuth cluster liquid metal ion gun (LMIG). The Bi_3_^2+^ primary ion beam was operated in a bunched mode to achieve high mass resolution. With a sample bias of approximately 3 kV, the secondary ions were extracted into a triple focusing time of flight (TRIFT) analyzer consisting of three electrostatic analyzers and then detected by a dual microchannel plate (DMCP) detector. Periportal and pericentral areas of 500 μm × 500 μm on the liver tissue sections were imaged with 256 × 256 pixels and a primary ion dose of approximately 2 × 1012 ions/cm^2^. Mass spectra were acquired over a mass range of *m/z* 0–1850 in both positive and negative polarities. Data processing was performed using PHI TOF-DR software. The mass spectra were internally calibrated using small fragment peaks, including C_2_H_3_^+^, C_3_H_5_^+^, and C_4_H_7_^+^ in positive ion mode and CH^−^, C_2_H^−^, and C_4_H^−^ in negative ion mode. Metabolite-specific fragments were assigned based on mass accuracy and previous studies [23,24], see Supplementary Table S3. ROI were manually defined in the TOF-DR software by comparing the ion images with stained images on adjacent tissue sections. The specific ion intensities of the amino acids extracted from the ROI were normalized to the regional total ion counts.

### 2.5 Enzymatic activity

GDH activity was measured from tissue extracts of liver and kidney lysed in 20 mM Tris–HCl (pH 8.0) with 2 mM CDTA and 0.2% Tween-20. GDH activity was detected in the oxidative deamination direction in 50 mM Tris–HCl buffer (pH 9.5, 2.6 mM EDTA, 1.5 mM NAD^+^, 1 mM ADP). The reaction was initiated by the addition of 4 mM glutamate and NADH autofluorescence was recorded over a 20 min period at 37 °C on a Fluostar Optima (BMG Labtech, Ortenberg, Germany). GDH enzymatic activity was calculated as initial velocity compared to standard curve obtained from the activity of purified liver GDH as detailed previously [25].

*In situ* enzymatic activities of GDH, succinate dehydrogenase (SDH) and glyceraldehyde-3-phosphate dehydrogenase (GAPDH) were assessed on liver cryosections by quantitative enzyme histochemistry using the nitro blue tetrazolium (NBT) assay as described previously [26,27]. Practically, when mice were sacrificed parts of their liver were embedded in OCT matrix (CellPath, Newtown, UK) and stored at −80 °C before analysis. Then, 7 μm cryosections were prepared and allowed to reach 37 °C in a dark humid chamber before addition of PBS buffer (0.1 M KH_2_PO_4_, 0.1 M Na_2_HPO_4_, pH 8.0 for mitochondrial GDH and SDH and pH 7.4 for cytosolic GAPDH) with 18% of polyvinyl alcohol (PVA, Alfa Aesar, Ward Hill, MA), 0.32 mM phenazine methosulfate (PMS, Sigma–Aldrich) for GDH and SDH and 0.45 mM 1-methoxy-PMS for GAPDH (mPMS, Interchim, Montluçon, France), 5 mM NBT (Toronto Chemical, Toronto, Canada), 1.0 mM ADP (Sigma–Aldrich) for GDH, 1.5 mM NAD^+^ (Applichem, Darmstadt, Germany) for GDH and GAPDH, and the respective specific substrates (4.0 mM glutamate for GDH, 0.6 mM succinate for SDH, and 2.5 mM glyceraldehyde-3-phosphate for GAPDH). After 20 min, reaction was stopped by washing the preparation with PBS at 60 °C, then with ultrapure water. Images were acquired under brightfield light on a Zeiss Axio Scan Z1 scanner using a 5× objective. For quantification of *in situ* activity, images were analysed using QuPath software and ROI were determined based on GS-positive versus GS-negative zonation according to the immunohistochemistry performed on subsequent sections from the same sample. For each section, absorbance values were measured on 5 different zones.

### 2.6 Statistical analysis

Statistical analyses were performed using GraphPad Prism 10.6.0 software. For more than two groups of data sets, two-way ANOVA was used for Bonferroni and Sidak multiple comparisons tests and Student's *t* test when only two groups of data were compared. The difference was considered significant when p < 0.05.

## 3 Results

### 3.1 Combined effects of dietary protein source and hepatic GDH on glucose control and circulating ammonia

**Figure 1:**
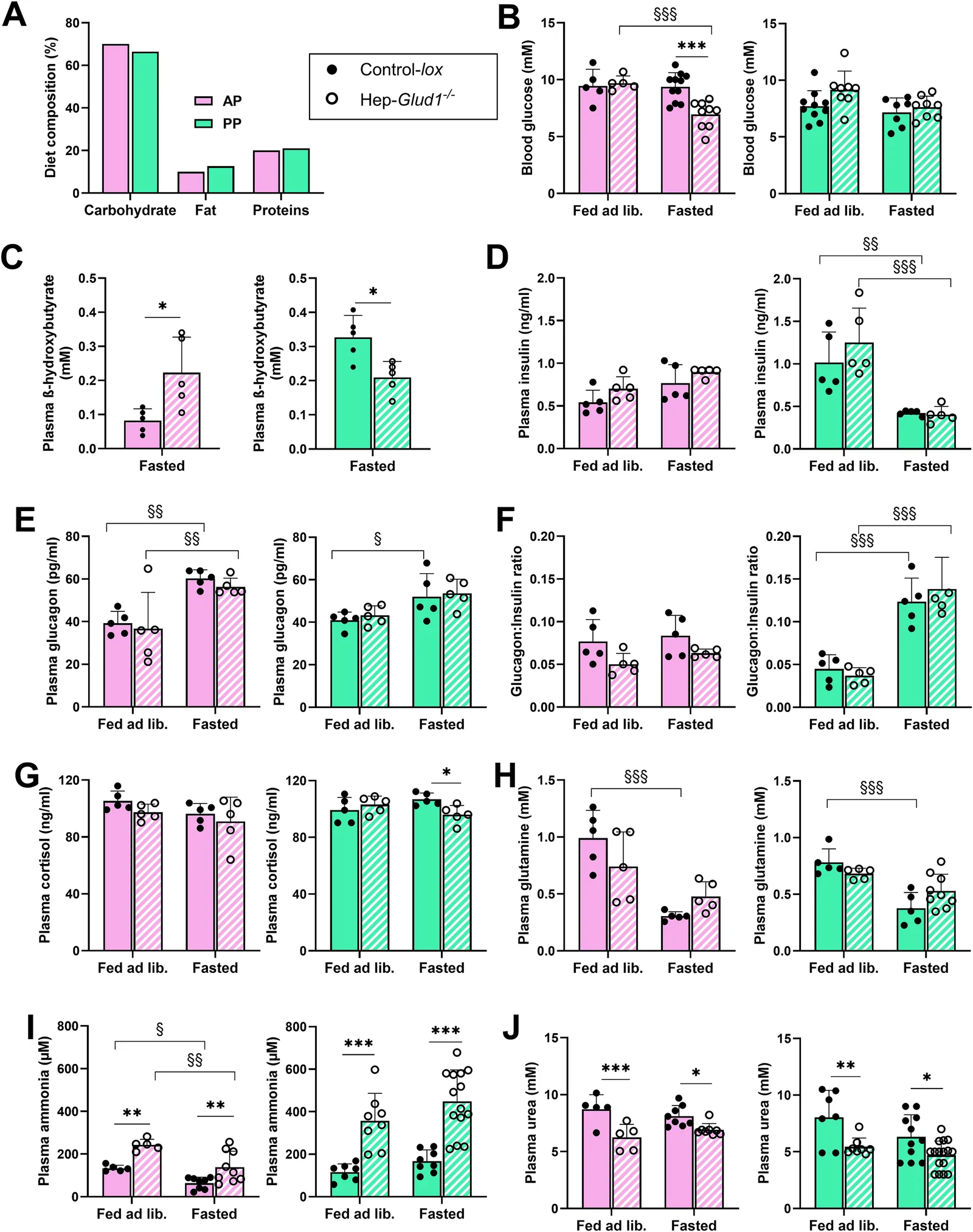
**Combined effects of dietary protein source and hepatic GDH on glucose control and circulating ammonia.** Blood was collected from Control-*lox* and Hep-*Glud1*^*−/−*^ mice mice fed ad libitum or fasted for 6 h after removal of their respective diets: animal-based (casein) proteins (AP, pink) or plant-based (cereals) proteins (PP, green). (**A**) Macronutrient contents of AP and PP diets (kcal%). (**B**) Blood glucose levels in fed and fasting states of mice under AP and PP diets. (**C**) Plasma β-hydroxybutyrate levels in 6 h fasted mice. Circulating hormonal levels in fed and fasted mice under AP and PP diets, respectively: insulin (**D**), glucagon (**E**), glucagon to insulin ratio (**F**), cortisol (**G**). Blood levels of the main circulating nitrogen carriers in fed and fasted mice under AP and PP diets, respectively: glutamine (**H**), ammonia (**I**), urea (**J**). Values are means ± SD, n = 5–16. Two-way ANOVA was used for multiple comparisons of genotypes and feeding states. ∗p < 0.05, ∗∗p < 0.01, ∗∗∗p < 0.001 versus corresponding feeding state; ^§^p < 0.05, ^§§^p < 0.01, ^§§§^p < 0.001 versus corresponding genotype.

Control mice (Control-*lox*) and mice lacking liver GDH (Hep-*Glud1*^*−/−*^) were fed diets with standard 20% (w/w) dietary protein contents. Proteins were from either animal (AP) or plant (PP) sources, *i.e*. casein (lactic, 30 Mesh) or cereals (mix of barley, wheat, maize, potato, sunflower) supplemented with brewer's yeast (rich in vitamin B complex), respectively. The rough nutrient composition of AP versus PP was: 70 vs. 66% carbohydrates, 10 vs. 13% fats, and 20 vs. 21% proteins, respectively (Figure 1A); with slight differences according to the specific ingredients composing each diet (Supplementary Table S1). Regarding amino acid composition, more than a 2-fold difference appeared for aspartate, cysteine, glycine, and tryptophan (2.62-, 2.54-, 2.41-, and 8.70-fold in PP versus AP diet), see Supplementary Table S4. Branched-chain amino acids were slightly lower in PP regimen (0.81- isoleucine, 0.81- leucine, and 0.91-fold valine in PP versus AP diet), as well as L-carnitine precursors (0.78- lysine, 0.69-fold methionine in PP versus AP diet). The levels of arginine were 1.69-fold higher in the PP versus AP diet and, as this amino acid carries four amino groups, such a difference in the plant diet might contribute to a relatively higher ammonia load. Glutamine content was similar between the two diets, although its precursor glutamate was 1.56-fold higher in the PP versus AP diet.

In the ad libitum fed state, blood glucose concentrations of control mice were lower when fed the PP diet than upon AP regimen (7.72 ± 1.36 versus 9.46 ± 1.46 mM, respectively, p = 0.040), and similar in mice knocked out for liver GDH (Figure 1B). Noteworthy, half of the carbohydrates in the AP diet was provided as sucrose, whereas unrefined cereals with lower glycemic index represent the only source in the PP diet (Supplementary Table S1). Fasting for 6 h, corresponding to physiological fasting [18,19], did not impact glycemia of control animals and in Hep-*Glud1*^*−/−*^ mice the lower levels conferred by the PP diet were not further reduced by the fasted state. However, knockout mice under AP diet did not maintain their higher blood glucose levels upon fasting (6.94 ± 1.19 mM, −28% versus fed Hep-*Glud1*^*−/−*^ mice, p < 0.001), which was associated with an energy stress response revealed by elevated β-hydroxybutyrate in knockout animals compared with controls (Figure 1C). The fasted state robustly reduced plasma insulin levels in both control and knockout PP mice, not in the AP groups (Figure 1D). Consequently, the HOMA-IR index used as a proxy of an euglycemic clamp [28], was markedly lower in control mice under PP versus AP diet (3.53 ± 0.49 versus 8.19 ± 1.93, respectively, p < 0.001); which was not further modified by the absence of liver GDH, in accordance with preserved glucose tolerance and insulin sensitivity in Hep-*Glud1*^*−/−*^ mice [9]. While glucagon globally increased in the fasting state, hormones controlling energy substrate availability (insulin, glucagon, cortisol) were not strikingly different between Control-*lox* and Hep-*Glud1*^*−/−*^ mice (Figure 1D–G), suggesting that changes in ketone bodies observed in the knockouts were not driven by hormonal responses. The need for endogenous glucose production increases the glucagon/insulin ratio [29]. This parameter was significantly increased by 6 h of fasting in mice fed the PP diet, while remaining in the range of the fed state in animals under the AP regimen (Figure 1F).

In mice, glycogen stores become depleted after about 6 h of fasting [18]. Then, continuation of endogenous glucose production can be prompted through gluconeogenesis at the expense of amino acids such as glutamine, which was reduced upon fasting in Control-*lox* mice, although not in Hep-*Glud1*^*−/−*^ mice (Figure 1H). Closely associated with amino acid dependent gluconeogenesis, liver GDH is required for active ornithine cycle and urea production that ensures safe disposal of ammonia [9]. While not observed upon the PP regimen, fasting after the AP diet significantly reduced circulating ammonia levels in both control and liver GDH knockout mice (Figure 1I). However, Hep-*Glud1*^*−/−*^ mice maintained higher ammonia and lower urea plasma levels compared to control mice (Figure 1I,J), pointing to impaired nitrogen detoxification in the absence of liver GDH. Overall, compared to the AP diet, ammonia homeostasis was worst when fasting after the PP diet, which resulted in significantly higher circulating ammonia levels, both in control (2.62-fold higher, p < 0.001) and in liver GDH knockout mice (3.22-fold higher, p < 0.001); while plasma urea levels were lower in PP versus AP groups (−22.3%, p = 0.027 for controls; −32.2%, p < 0.001 for knockouts).

Collectively, these results reveal specific adaptations to dietary protein sources with hepatic GDH playing a key role in the maintenance of energy substrate distribution. The lack of liver GDH combined with the PP diet resulted in severe hyperammonemia.

### 3.2 Hepatic GDH controls circadian energy substrate utilization and food intake according to the dietary protein source

The effects of dietary protein sources and liver GDH on the glucose balance suggested adaptive responses to animal versus plant-based diets. To explore the transition from one to the other diet, we evaluated food consumption and corresponding calorie intake in mice fed with the PP diet, then switched to AP regimen for 4 days and back to PP diet for another 4-day period. When diet was changed from PP to AP, mice reduced their daily food intake with a regain once back on the PP diet (Supplementary Fig. S1A–D). This was observed in both Control-*lox* and Hep-*Glud1*^*−/−*^ mice. As reported previously [9], the pattern of day/night food intake was shifted in Hep-*Glud1*^*−/−*^ mice compared to Control-*lox* under the PP diet. However, the shift contributed by the absence of liver GDH was nearly relieved when mice were fed the AP diet, revealing specific responses according to the protein sources and a rapid adaptation to food composition (Supplementary Fig. S1E and F).

**Figure 2:**
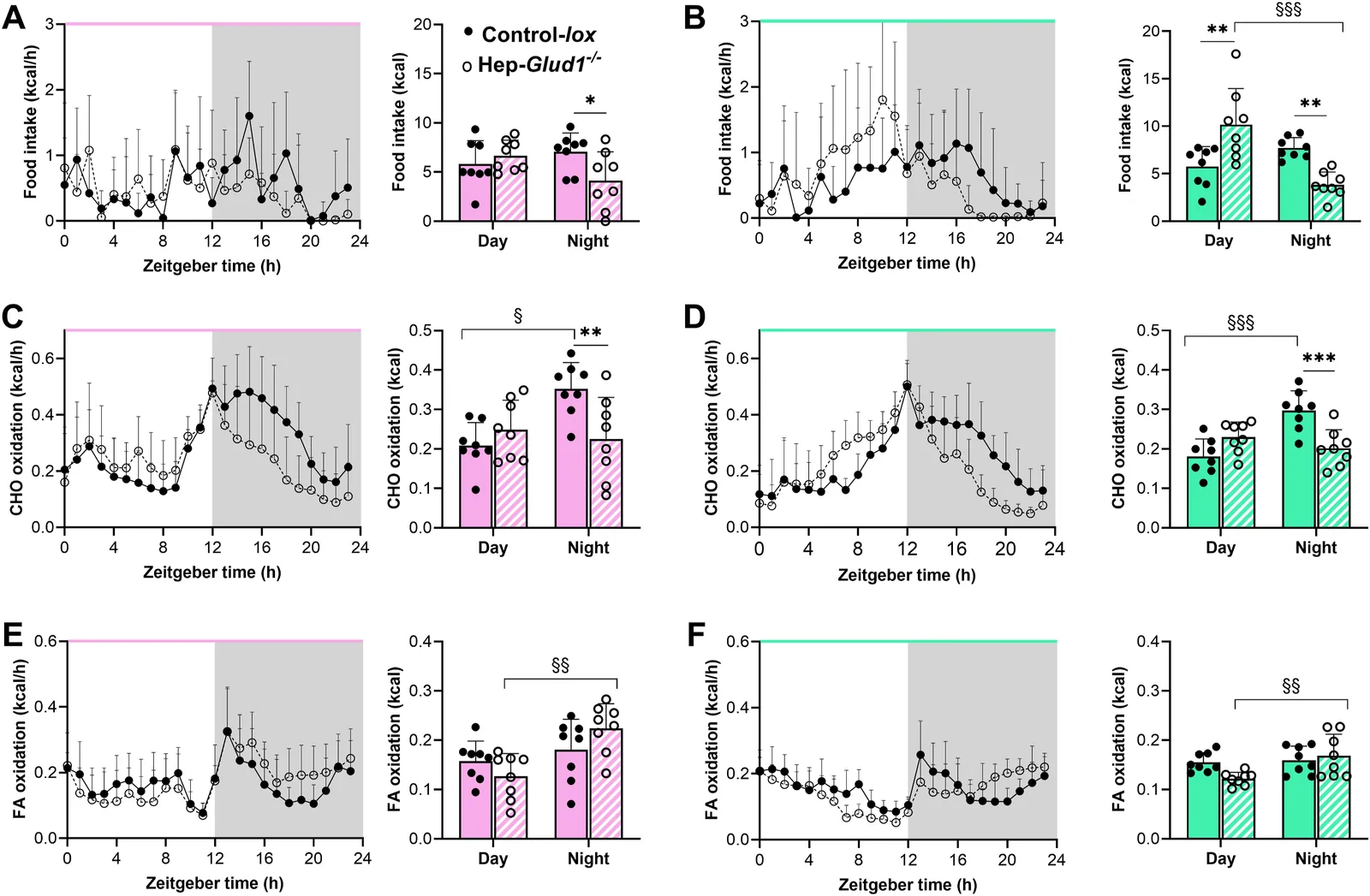
**Effects of hepatic GDH on circadian substrate utilization and food intake according to the dietary protein source**. Metabolic parameters of Control-*lox* and Hep-*Glud1*^*−/−*^ mice fed diets composed of either animal-based proteins (pink) or plant-based proteins (green) were recorded over 4 consecutive periods of 24 h. The last 24 h period of recording is shown with day and night quantifications. (**A**, **B**) Food intake (kcal); (**C**, **D**) carbohydrate (CHO) oxidation; (**E**, **F**) fatty acid (FA) oxidation. AUC was calculated according to the Zeitgeber day and nighttime. Values are means ± SD, n = 8. Two-way ANOVA was used for Bonferroni and Sidak multiple comparisons tests of the genotypes of mice and dietary protein sources. ∗p < 0.05, ∗∗p < 0.01, ∗∗∗p < 0.001 versus the corresponding Zeitgeber period; §p < 0.05, §§p < 0.01, §§§p < 0.001 versus the corresponding genotype.

Further analysis of the circadian rhythm showed that under the AP diet the food intake of Hep-*Glud1*^*−/−*^ was reduced at night compared to Control-*lox* (Figure 2A). When fed the PP diet, the phenotype of Hep-*Glud1*^*−/−*^ mice was similar, although the effects of a lack of hepatic GDH on eating behavior were exacerbated with (i) increased food intake at Zeitgeber time 7 h (ZT7, 2:00 pm) and (ii) reduced consumption from ZT18 to ZT21 (Figure 2B). This anticipated food intake by Hep-*Glud1*^*−/−*^ mice in the daytime before lights off is indicative of impaired endogenous glucose production in the fasting state, required by the PP diet but not upon AP regimen. As expected for control mice, eating mostly in the dark phase was associated with enhanced carbohydrate utilization at night. However, mice lacking liver GDH exhibited an alteration in nocturnal energy substrate utilization with reduced carbohydrate oxidation (Figure 2C–F), not efficiently compensated by fatty acid catabolism when fed the PP diet as corresponding animals had reduced fat oxidation at night (−75.1% versus AP fed Hep-*Glud1*^*−/−*^ mice, p = 0.0310).

These results highlight the critical role of hepatic GDH in bridging energy substrate utilization and feeding behavior rhythms, highlighted by the response specificities to different dietary protein sources.

**Figure 3:**
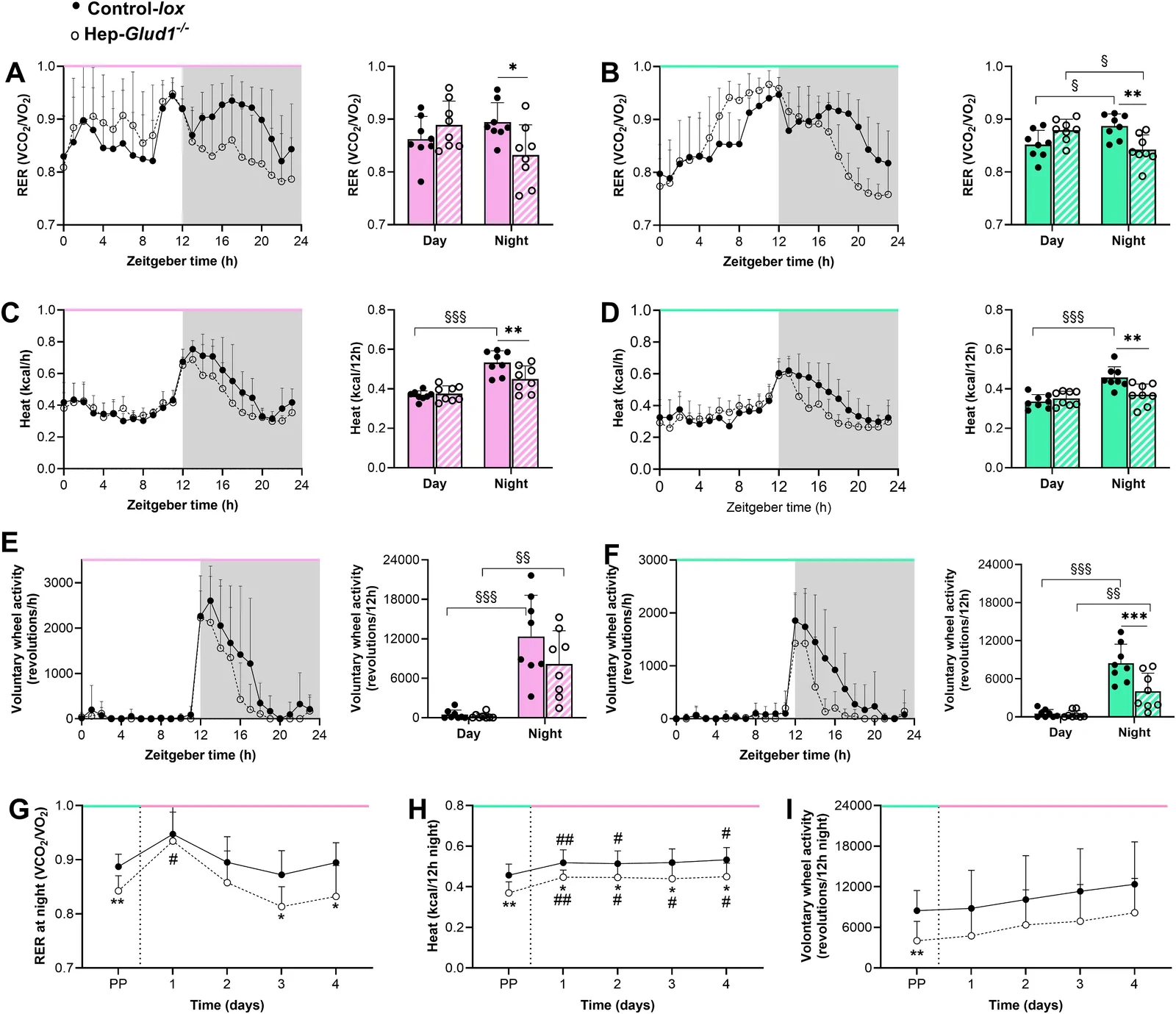
**Effects of liver GDH on circadian energy expenditure and physical activity according to the dietary protein source**. Metabolic and activity parameters of Control-*lox* and Hep-*Glud1*^*−/−*^ mice fed diets composed of either animal-based proteins (AP, pink) or plant-based proteins (PP, green) were recorded over 4 days and (**A-F**) the last 24 h period is shown with day and night quantifications. (**A**, **B**) RER (VCO_2_/VO_2_); (**C**, **D**) heat production; (**E**, **F**) voluntary activity assessed by free running wheel. AUC was calculated according to the Zeitgeber day and nighttime. (**G-I**) Switch from PP to AP diet followed over 4 days; (**G**) RER at night, (**H**) heat production at night, (**I**) voluntary activity at night assessed by free running wheel. Values are means ± SD, n = 8. Two-way ANOVA was used for Bonferroni and Sidak multiple comparisons tests of the genotypes of mice and dietary protein sources. ∗p < 0.05, ∗∗p < 0.01, ∗∗∗p < 0.001 versus the corresponding time phase; §p < 0.05, §§p < 0.01, §§§p < 0.001 versus the corresponding genotype; #p < 0.05, ##p < 0.01 versus PP period.

Considering the observed changes in energy expenditure, we next assessed the respiratory exchange ratio (RER) as an indicator of the continuous balance between carbohydrate and lipid oxidation. During the dark phase, under both AP and PP diets, Hep-*Glud1*^*−/−*^ mice had reduced VCO_2_ production (Supplementary Fig. 2A and B) and exhibited a lower RER compared to Control-*lox*, underlying higher lipid expenditure in the knockouts (Figure 3A,B). This was accompanied by decreased nocturnal heat production in Hep-*Glud1*^*−/−*^ mice versus corresponding Control-*lox* animals fed the same diet (Figure 3C,D), while under the PP diet control mice dissipated less energy (−14.2% versus AP fed Control-*lox*, p = 0.0196). Regarding locomotor activity, although total movement counts were not significantly different between day and night in all groups (Supplementary Fig. 2C–F), Control-*lox* mice experienced high spontaneous physical activity during the dark phase, as revealed by the free running wheel (Figure 3E,F). This voluntary activity was significantly reduced in Hep-*Glud1*^*−/−*^ mice under the PP regimen. Interestingly, this was observed even though Hep-*Glud1*^*−/−*^ mice ate less at night, indicating decreased global activity in knockout animals that limited energy waste, as reflected by reduced heat production. Overall, PP diet combined with liver GDH knockout exhibited strong additive alteration of spontaneous physical activity with a 67.4% (p = 0.0041) lower running activity compared with control mice fed the AP diet.

To test if the lower activity associated with the PP diet in the absence of hepatic GDH could be regained by switching to the AP diet, we next monitored nocturnal parameters over a period of 4 days on AP diet after stabilization on PP regimen. RER was transiently elevated and heat production increased in both Control-*lox* and Hep-*Glud1*^*−/−*^ mice, while keeping the differences between genotypes (Figure 3G,H). Interestingly, switching from PP to AP diet rapidly raised voluntary physical activity, resulting in the loss of significant differences between control and knockout animals (Figure 3I).

### 3.3 Role of hepatic GDH and circulating amino acids in the gluconeogenic capacity upon fasting

**Figure 4:**
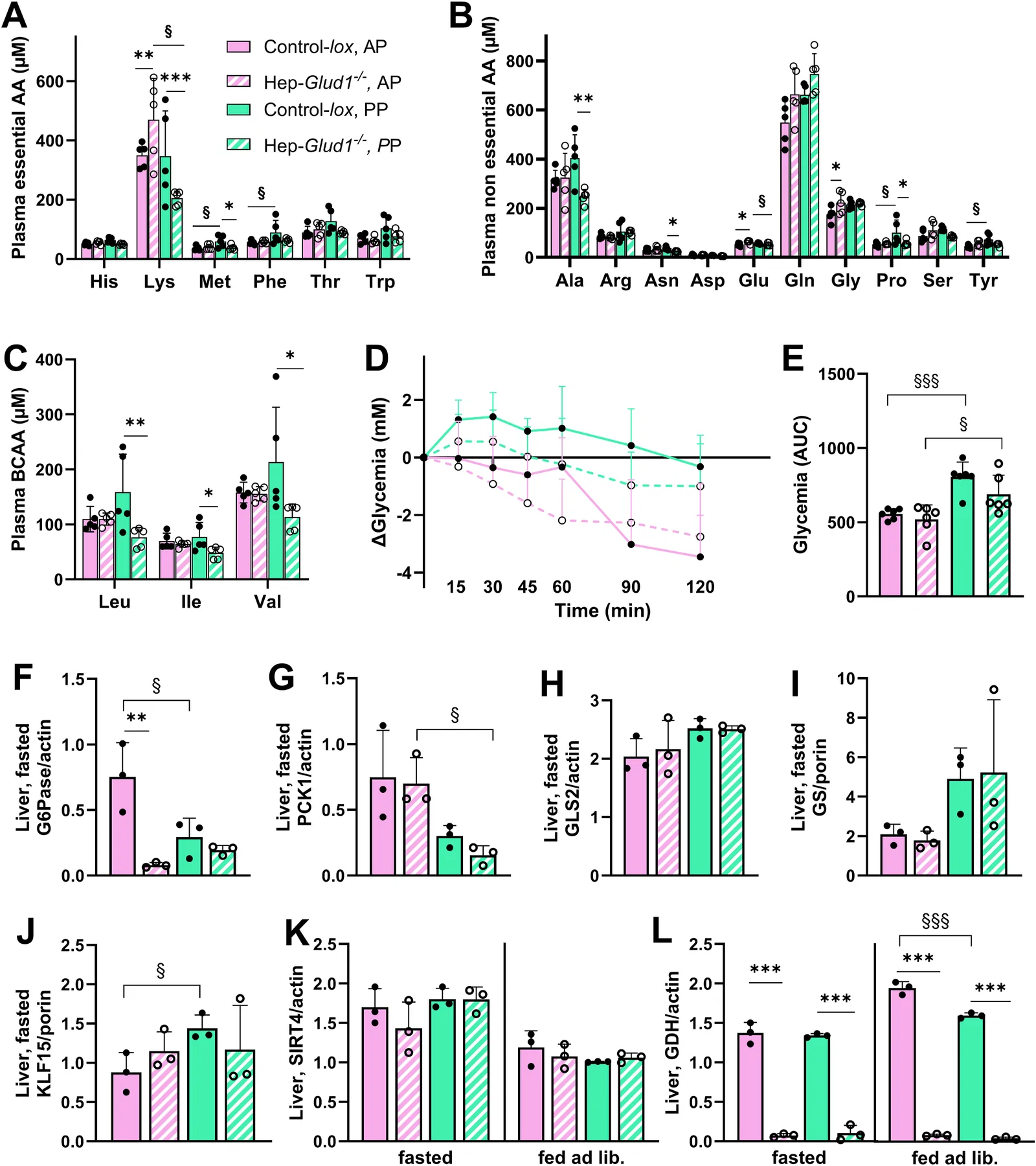
**Role of hepatic GDH and circulating amino acids in the gluconeogenic capacity upon fasting.** Control-*lox* and Hep-*Glud1*^*−/−*^ mice mice fed diets composed of either animal-based proteins (AP, pink) or plant-based proteins (PP, green) were fasted for 6 h before analyses. (**A**–**C**) Plasma levels of amino acids: essential (**A**), non-essential (**B**), and essential branched-chain BCAA (**C**); n = 5. (**D**, **E**) Blood glucose concentrations over 2 h after an i. p. Injection of 1 g/kg alanine plus 1 g/kg glutamine (**D**) and corresponding AUC quantification (**E**); n = 5–6. (**F**–**I**) Liver protein levels assessed by immunoblotting of: (**F**) glucose-6-phosphatase (G6Pase), (**G**) phosphoenolpyruvate carboxykinase 1 (PCK1), (**H**) glutaminase 2 (GLS2), (**I**) glutamine synthetase (GS), (**J**) Krüppel-like factor 15 (KLF15), and (**K**) sirtuin SIRT4, (**L**) GDH; n = 3. Values are means ± SD. Two-way ANOVA was used for Bonferroni and Sidak multiple comparisons tests of the genotypes of mice and dietary protein sources. ∗p < 0.05, ∗∗p < 0.01, ∗∗∗p < 0.001 versus the corresponding dietary protein sources; ^§^p < 0.05, ^§§^p < 0.01, ^§§§^p < 0.001 versus the corresponding genotype.

Considering the impact of lower carbohydrate usage in Hep-*Glud1*^*−/−*^ mice, we next evaluated the availability of the protein-derived substrates required for *de novo* glucose production, as well as the capacity of the liver to make use of gluconeogenic amino acids. Blood concentrations of amino acids were measured by LC-MS in plasma samples collected from 6 h fasted mice. In Control-*lox* animals, circulating levels of essential amino acids were comparable when mice were fed either AP or PP diets, although methionine and phenylalanine were increased under the plant-based diet (Figure 4A). Similar marginal effects were observed for non-essential amino acids proline and tyrosine (Figure 4B). Under the AP diet, the lack of hepatic GDH increased plasma levels of lysine, glycine, and glutamate. Strikingly, several amino acids were lower in plasma of knockout mice fed with the PP diet compared to control mice under the same regimen: L-carnitine precursors lysine and methionine, asparagine, proline, and all the essential branched-chain amino acids, as well as gluconeogenic alanine (−38.7%, p = 0.0071); see Figure 4A–C.

Because alanine and glutamine are the main amino acids contributing to hepatic gluconeogenesis [30], *in vivo* blood glucose changes were measured in response to an acute administration of a mix of alanine and glutamine in 6 h fasted mice. As shown above (Figure 1B), mice on a PP diet exhibited lower blood glucose levels than those fed the AP diet. Interestingly, only PP-fed Control-*lox* animals showed effective amino acid-induced blood glucose elevations, raising their glycemia above their respective basal fasting levels (Figure 4D,E). Consistent with previous reports, this was not operative in Hep-*Glud1*^*−/−*^ mice [9].

Key enzymes for gluconeogenesis were then assessed by immunoblotting in liver extracts (immunoblots shown on Supplementary Fig. S3). Under the fasted state, glucose-6-phosphatase (G6Pase) showed the highest levels in Control-*lox* mice fed the AP diet, while phosphoenolpyruvate carboxykinase 1 (PCK1) was the lowest in Hep-*Glud1*^*−/−*^ mice under PP regimen (Figure 4F,G). Hepatic glutaminase 2 (GLS2) and glutamine synthetase (GS), controlling glutamine breakdown and synthesis from glutamate, respectively, were not significantly changed by the different conditions (Figure 4H,I). The fasting-induced transcription factor Krüppel-like factor 15 (KLF15), a key regulator of gluconeogenesis [31], was upregulated in Control-*lox* mice fed the PP diet versus AP regimen (Figure 4J). We observed no significant changes for the mitochondrial sirtuin SIRT4 that inhibits GDH through NAD^+^-dependent ADP-ribosyltransferase activity [32], under both fasted and fed states (Figure 4J). As expected, GDH was absent in the liver of Hep-*Glud1*^*−/−*^ mice and at similar levels in fasted Control-*lox* under either diet (Figure 4K). However, lower GDH expression was observed in ad libitum fed Control-*lox* mice under PP versus AP diet. Protein levels of N-acetyl-glutamate synthase (NAGS), that participates in ammonia handling and urea formation, were not significantly altered by the lack of liver GDH (Supplementary Fig. S4A). Under fed conditions, the relative pattern of expression among groups was similar to the fasting state, except for GLS2 and GS being up and down regulated respectively in Hep-*Glud1*^*−/−*^ mice eating PP diet (Supplementary Fig. S4B–E). As the kidney also participates to gluconeogenesis [33], we checked the expression of GDH and some relevant enzymes (Supplementary Fig. 4F–J) showing no change in GDH but lower levels of G6Pase under PP versus AP diet and upregulation of kidney glutaminase 1 (GLS1) in Hep-*Glud1*^*−/−*^ mice fed the AP regimen.

**Figure 5:**
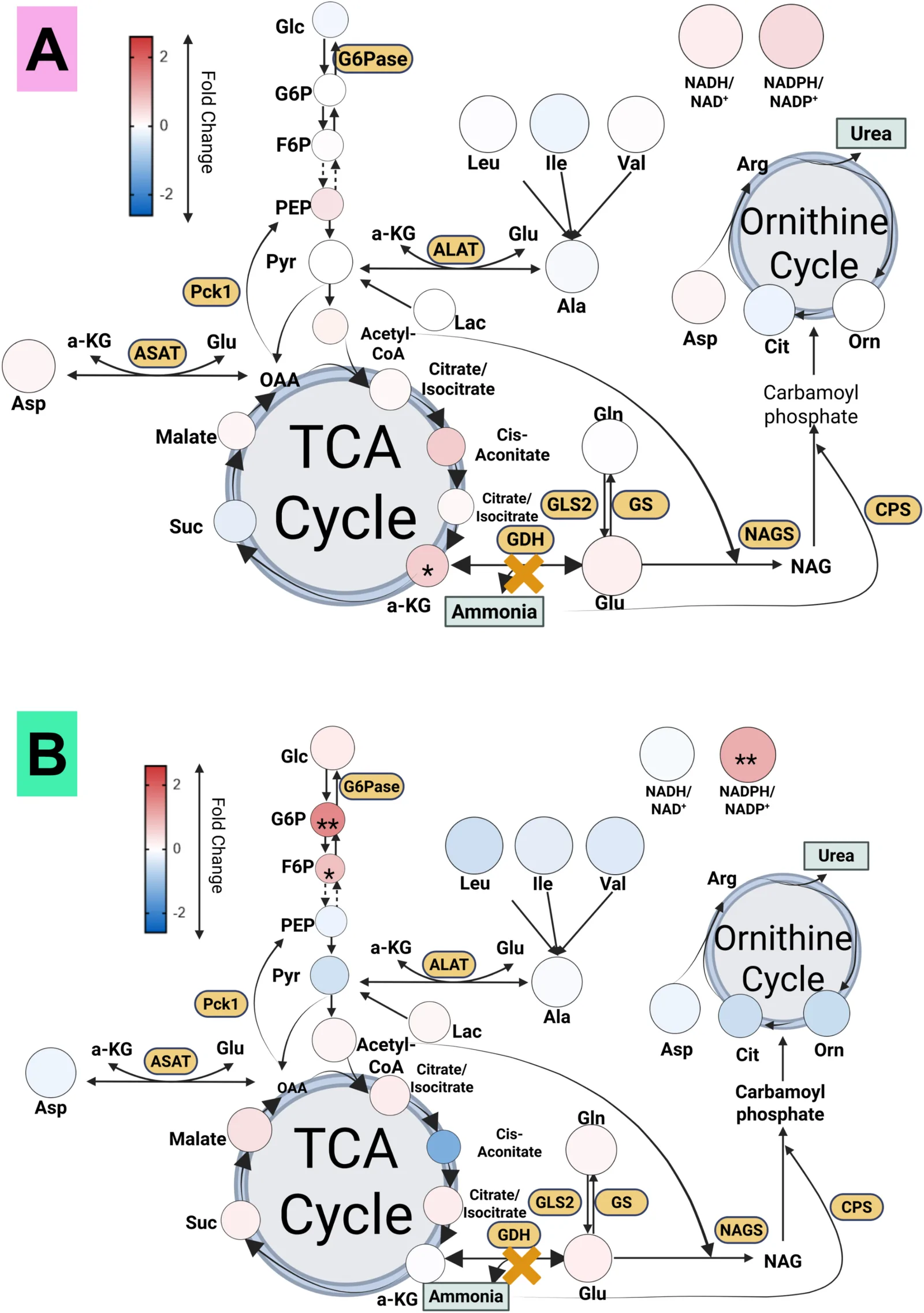
**Role of hepatic GDH according to the dietary protein source on liver metabolite levels.** Control-*lox* and Hep-*Glud1*^*−/−*^ mice mice fed diets composed of either animal-based proteins (AP, pink) or plant-based proteins (PP, green) were fasted for 6 h before analyses. (**A**, **B**) Liver metabolites measured on liver extracts and presented as schemes of biochemical pathways for glycolysis, the TCA cycle, and the ornithine cycle (see Supplemental Tables S5 and S6 for actual means ± SD values). Each circle shows the relative levels of metabolites in Hep-*Glud1*^−/−^ versus Control-*lox* mice fed either AP (**A**) or PP (**B**) diets. Relative values range from −2.5 (blue) to +2.5 (red) and significant differences are indicated in the circle; n = 5. Two-way ANOVA was used for Bonferroni and Sidak multiple comparisons tests of the genotypes of mice and dietary protein sources. ∗p < 0.05, ∗∗p < 0.01 versus the corresponding dietary protein sources.

Next, the levels of metabolites in liver extracts of 6 h fasted mice were determined by LC-MS. In Control-*lox* animals, compared to the PP diet, AP diet was associated with lower levels of key intermediates used in both gluconeogenesis (acetyl-CoA, phosphoenolpyruvate, and NADH/NAD^+^ ratio) and the ornithine cycle (citrulline, see Supplementary Fig. S5A and Tables S5 and S6), suggesting faster exhaustion of these elements in mice undergoing fasting after AP versus PP feeding. In Hep-*Glud1*^*−/−*^ mice, the difference between the two diets was mostly seen in the upstream and downstream intermediates of gluconeogenesis, with respectively higher pyruvate and lower fructose-6-P and glucose-6-P (Supplementary Fig. S5B). This indicates that, in the absence of hepatic GDH, pyruvate-derived gluconeogenesis was less solicited in fasted mice after an AP regimen than a PP diet. When evaluated based on identical dietary protein source, Hep-*Glud1*^*−/−*^ mice on AP diet exhibited similar liver metabolite profile as compared to Control-*lox* animals on the same food (Figure 5A), except for higher α-ketoglutarate in knockouts. However, after being fed the PP diet, fasting Hep-*Glud1*^*−/−*^ mice produced markedly more fructose-6-P and glucose-6-P than Control-*lox* mice (Figure 5B), pointing to alternative substrates being used to compensate for defective amino acid-induced gluconeogenesis. Accordingly, L-carnitine precursor lysine required for β-oxidation was markedly reduced in the liver of Hep-*Glud1*^*−/−*^ mice under the PP diet (Supplemental Table S6).

Collectively, the expression profile indicates higher enzymatic capacity for gluconeogenesis under the AP diet, which is blunted by liver GDH knockout but compensated by increased fat expenditure. Metabolomics revealed specific adaptations to the lack of hepatic GDH according to the source of dietary proteins.

### 3.4 Effects of the dietary protein source on hepatic GDH activity in fed and fasting states

**Figure 6:**
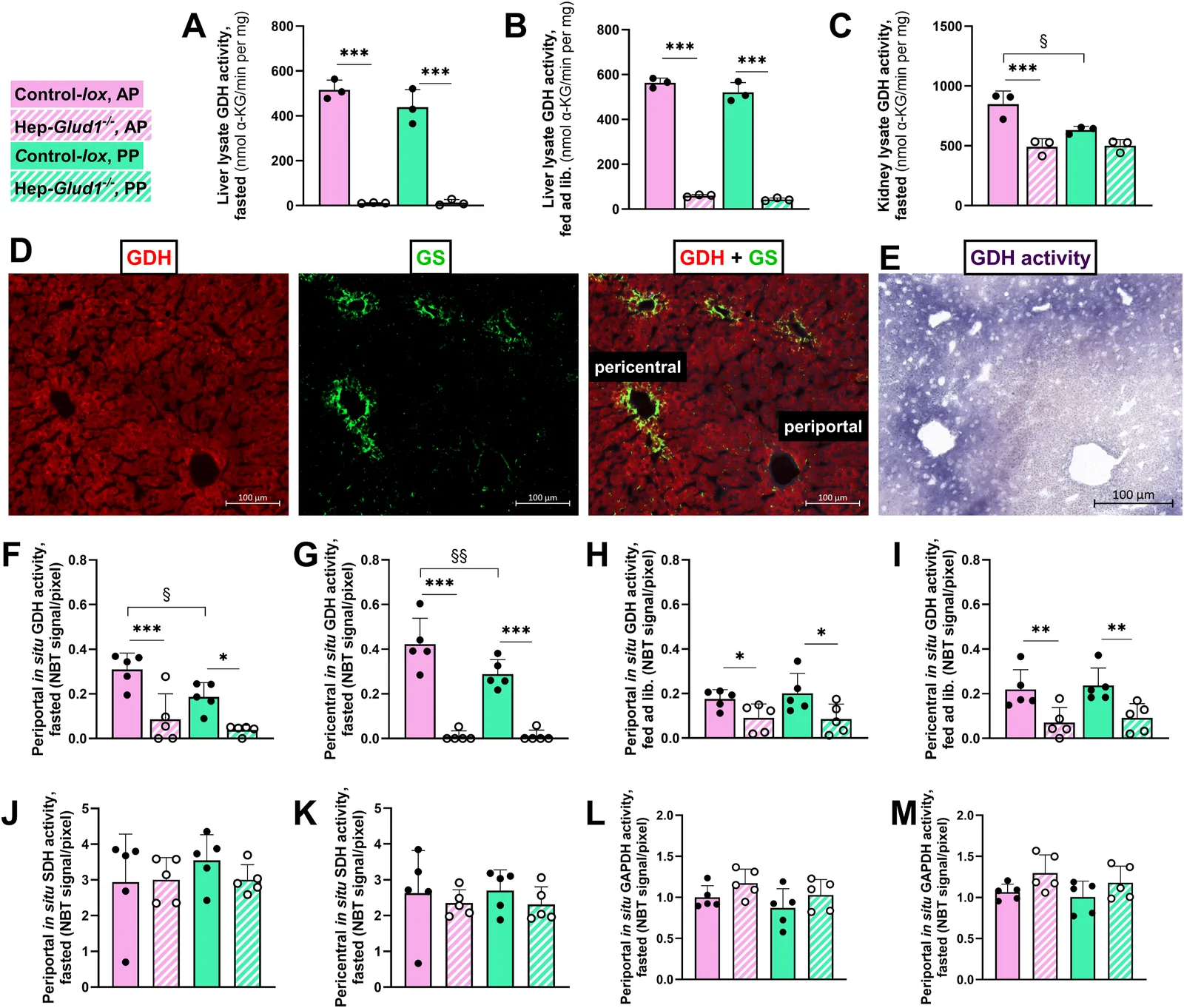
**Effects of the dietary protein source on hepatic GDH activity in fed and fasting states.** Liver and kidney of Control-*lox* and Hep-*Glud1*^*−/−*^ mice mice fed diets composed of either animal-based proteins (AP, pink) or plant-based proteins (PP, green) were collected in both ad libitum fed state and following 6 h of fasting before analyses. (**A**–**C**) GDH activity measured in lysates of liver from fasted (**A**) and fed (**B**) mice, and of kidney of fasted animals (**C**). (**D**, **E**) Representative immunoreactive staining for GDH and GS (**D**), and *in situ* GDH enzymatic activity (**E**) performed on sequential liver cryosections of Control-*lox* fed AP diet; scale bar = 100 μm. (**F**–**I**) Quantification of liver *in situ* GDH activity of fasted mice in periportal (**F**) and pericentral (**G**) regions and of fed mice in the periportal (**H**) and pericentral (**I**) regions. (**J-K**) Liver *in situ* succinate dehydrogenase (SDH) activity of fasted mice in periportal (**J**) and pericentral (**K**). (**L-M**) Liver *in situ* glyceraldehyde-3-phosphate dehydrogenase (GAPDH) activity of fasted mice in periportal (**L**) and pericentral (**M**). Values are means ± SD, n = 3–5. Two-way ANOVA was used for multiple comparisons of the genotypes of mice and dietary protein sources. ∗p < 0.05, ∗∗p < 0.01, ∗∗∗p < 0.001 versus the corresponding dietary protein sources; ^§^p < 0.05, ^§§^p < 0.01 versus the corresponding genotype.

GDH enzymatic activity measured in liver lysates exhibited similar velocity in Control-*lox* mice fed either AP or PP diets, both in the fed state and following 6 h of fasting (Figure 6A,B), while some residual activity was detectable in Hep-*Glud1*^*−/−*^ mice. In the kidney, GDH activity in fasted Control-*lox* mice was higher under the AP diet versus the PP diet (Figure 6C). Interestingly, liver GDH knockout reduced kidney GDH function of AP-fed Hep-*Glud1*^*−/−*^ mice to velocity similar to control mice under the PP regimen. Thus, renal GDH activity was not directly associated with blood ammonia levels, while chronic hyperammonemia may lead to oxidative stress on the kidney [34].

Hepatic GDH activity is not evenly distributed throughout the liver despite homogenous expression levels [35]. This was shown by immunohistochemistry in liver sections of Control-*lox* mice fed the AP diet using GS staining to reveal pericentral regions as opposed to GS-negative periportal zones (Figure 6D). Performed on the subsequent liver cryosections, *in situ* GDH activity was more robust in pericentral versus periportal regions (Figure 6E). Corresponding quantification of liver *in situ* GDH function revealed impacts of the dietary protein source with higher periportal and pericentral GDH activity in fasted Control-*lox* mice under the AP diet versus the PP diet (Figure 6F,G). Compared to fasting, the fed state resulted in lower GDH activity with both diets (Figure 6H,I). Assessed as flagships of respectively mitochondrial and glycolytic capacity, SDH and GAPDH activities were modified neither by the diet nor by the lack of liver GDH (Figure 6J-M). Of note, mitochondrial morphology of Hep-*Glud1*^*−/−*^ hepatocytes is preserved [9].

This set of data shows region-specific GDH activity in the liver and adaptation to the source of dietary proteins resulting in stronger GDH function in the fasting state following AP versus PP feeding.

### 3.5 *In situ* metabolomics discriminates between periportal and pericentral changes contributed by diet and GDH

**Figure 7:**
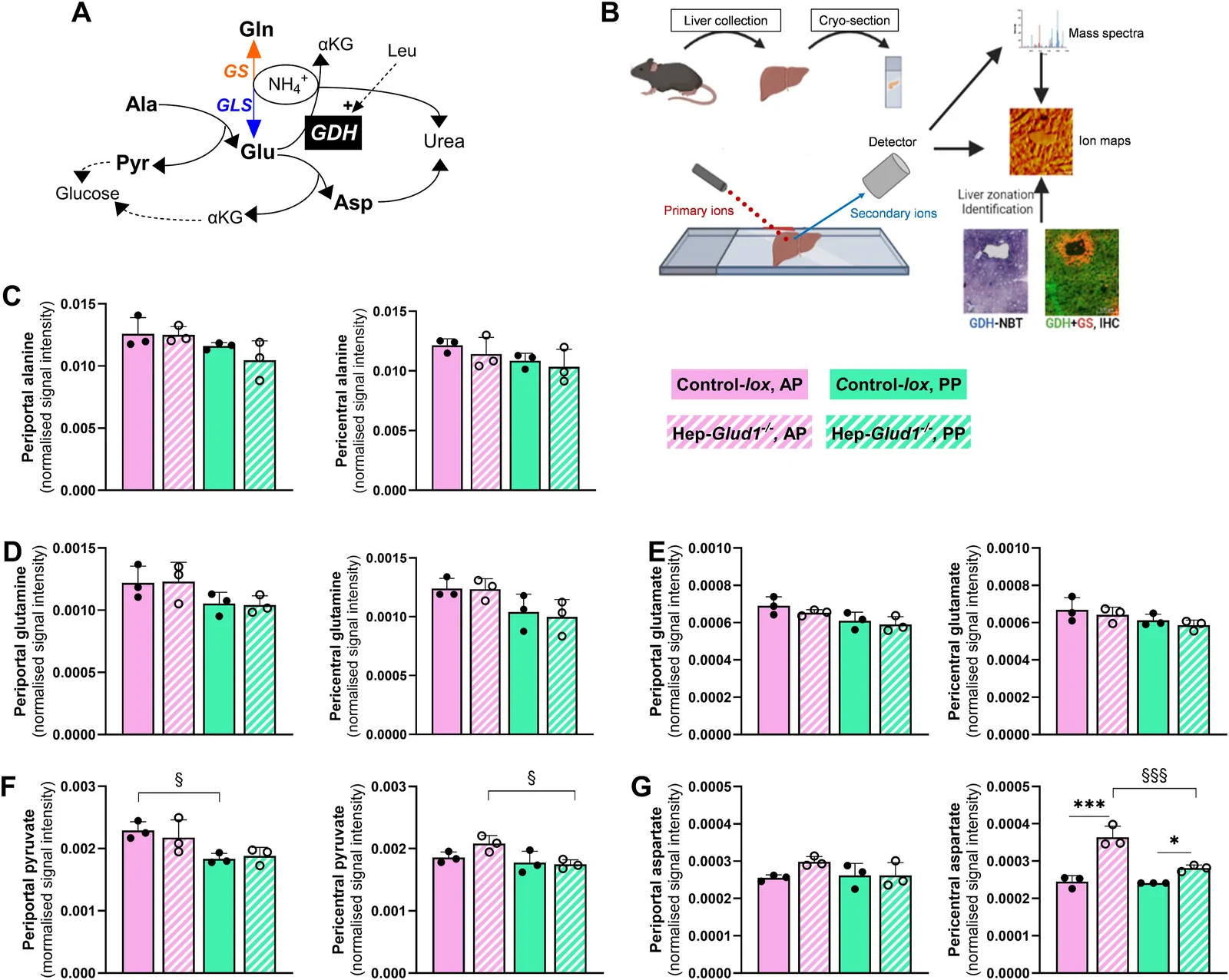
**Effects of the dietary protein source and hepatic GDH on liver metabolite zonation.** Control-*lox* and Hep-*Glud1*^*−/−*^ mice mice fed diets composed of either animal-based proteins (AP, pink) or plant-based proteins (PP, green) were fasted for 6 h before analyses. (**A**) Main GDH-associated pathways and dominant fluxes according to liver zonation with key metabolites relevant for gluconeogenesis and urea formation (Gln, glutamine; Ala, alanine; Asp, aspartate; Pyr, pyruvate; Glu, glutamate; αKG, α-ketoglutarate; *GLS*, glutaminase, predominantly in periportal hepatocytes, blue arrow; *GS*, glutamine synthetase, exclusively in pericentral hepatocytes, orange arrow). (**B**) General scheme of the ToF-SIMS experimental procedure. Immunohistochemistry performed on sequential liver cryosections was used to identify regions of interest in the periportal and pericentral regions, respectively. Periportal and pericentral levels of (**C**) alanine, (**D**) glutamine, (**E**) glutamate, (**F**) pyruvate, (**G**) aspartate. Values are means ± SD, n = 3. Two-way ANOVA was used for multiple comparisons of the genotypes of mice and dietary protein sources. ∗p < 0.05, ∗∗∗p < 0.001 versus the corresponding dietary protein sources; ^§^p < 0.05, ^§§§^p < 0.001 versus the corresponding genotype.

According to the importance of hepatic zonation and the specific contributions of periportal versus pericentral regions in gluconeogenesis and urea formation, we next performed spatial *in situ* metabolomics using high resolution ToF-SIMS imaging on liver cryosections (Figure 7A,B). By using a highly focused ion beam to raster across a defined sample area pixel by pixel, ToF-SIMS records mass spectra from each pixel position and provides ion maps showing the spatial distribution of the detected metabolites.

Immunohistochemistry performed on sequential liver cryosections was used to identify regions of interest in the periportal and pericentral regions, respectively (Supplementary Fig. S6). Metabolite-specific fragments were assigned based on previous reports [23,24] as well as mass accuracy, namely by comparing the theoretical mass to the mass to charge (*m/z*) ratio in the ToF-SIMS spectra in positive and negative modes (Supplementary Figs. S7–S9). Levels of the gluconeogenic substrates alanine and glutamine, as well as glutamate levels, were not statistically different among groups (Figure 7C–E). However, the gluconeogenic intermediate pyruvate was lower under the PP versus the AP diet in periportal regions of Control-*lox* mice and in pericentral regions of Hep-*Glud1*^*−/−*^ animals (Figure 7F). Regarding urea formation, pericentral aspartate levels were increased by GDH knockout under both diets, while being lower in Hep-*Glud1*^*−/−*^ mice fed PP versus AP diet (Figure 7G).

These results uncover region-specific differences in the levels of key metabolites required for both glucose and urea production.

## 4 Discussion

Slight differences in food composition, such as animal AP versus plant PP protein sources, may potentially impact energy substrate availability and spontaneous physical activity. In the present study, based on equivalent dietary proportions of carbohydrate, fat and proteins, we investigated the short-term adaptation to AP versus PP diets and the respective outcomes regarding glucose control and energy expenditure. Because the ingredients used to compose AP and PP diets rely on different protein sources, i.e. casein versus cereal mix respectively, the carbohydrate profile differed with higher sucrose content in the AP diet compared to PP regimen. At least in the fed state, this should result in higher glycemic index, which was witnessed in the present study through lower glycemia in ad libitum fed mice under PP versus AP diet. Regarding amino acids, one of the main differences between the two diets with similar 20% protein contents was the lower content of branched-chain amino acids in the PP diet versus the AP regimen and about 9-fold higher tryptophan content in PP compared to AP diet. Considering the dietary branched-chain amino acids, their reduction for a period of 5 weeks in mice was shown to lower fasting blood glucose and circulating insulin levels [36].

In this work, we made similar observations regarding the fasting state, while effects were observed already after 4 days of reduced intake of branched-chain amino acids under the plant-based regimen. This was associated with significantly lower HOMA-IR index compared to the AP diet, in agreement with increased insulin sensitivity in mice upon reduction of dietary branched-chain amino acids [36]. Upon feeding, the branched-chain amino acid leucine can activate mTOR complex 1 (mTORC1) to promote protein synthesis, while being inhibited during fasting periods [37,38]. As observed upon fasting, in the fed state mice under the AP diet exhibited higher glycemia compared to the PP regimen, although insulin levels were lower in the AP group. Per se, the lack of liver GDH did not alter glucose homeostasis in fed mice but resulted in higher circulating ammonia and lower urea levels, as observed previously [35].

Upon physiological 6 h fasting, we observed differences in ammonia homeostasis in control mice under PP versus AP regimens. Measured *in situ* to preserve liver zonation, GDH activity was lower in the periportal region of the PP group, while hepatic levels of citrulline were higher compared to AP controls. This indicates that GDH-mediated provision of ammonia to the ornithine cycle was less efficient in the PP group promoting the buildup of its intermediate citrulline, ultimately reducing urea synthesis. Indeed, plasma levels of urea were lower in mice under the PP diet with elevated circulating ammonia. Interestingly, spatial metabolomics showed accumulation of aspartate in pericentral regions of Hep-*Glud1*^*−/−*^ mice versus controls, suggesting lower usage of this key metabolite for the ornithine cycle in the upstream periportal region in charge of the urea formation. The ensuing increased plasma ammonia levels in Hep-*Glud1*^*−/−*^ animals were much more pronounced under the PP diet compared to AP regimen, showing synergistic effects between PP diet and liver GDH knockout, resulting in severe hyperammonemia in Hep-*Glud1*^*−/−*^ mice under plant-based proteins. This was associated with markedly lower spontaneous physical activity, eventually recapitulating some form of ataxia comparable to those observed in ammonia brain toxicity [39] triggered by hepatic encephalopathy [40]. The lower nocturnal activity was accompanied by reduced food intake and altered energy partitioning, *i.e*. lower carbohydrate consumption and increased lipid oxidation. Because Hep-*Glud1*^*−/−*^ mice cannot efficiently run hepatic gluconeogenesis from amino acids [9], the change in nutrient source for energy expenditure might also be contributed by limitation in hepatic glucose production. Constraint in energy expenditure in Hep-*Glud1*^*−/−*^ mice was further exacerbated in animals fed the PP diet with limited availability of lysine serving as a necessary precursor for L-carnitine synthesis required for mitochondrial β-oxidation of fatty acids, when not provided by dietary meat [41]. These results highlight the key function of liver GDH in the maintenance of ammonia homeostasis, in particular when dietary proteins are essentially from plant-based source, impacting whole body energy expenditure as well as behavior.

During fasting, glucose supply from the digestive track becomes progressively limiting. The response of the organism aims at preserving sugar for glucose-dependent organs with simultaneous glucose production by the liver through glycogenolysis, followed by gluconeogenesis [42]. Compared to mice fed the AP diet, animals under PP diet exhibited a more robust fasting response with significant elevation of the glucagon/insulin ratio and upregulation of liver KLF15, a critical regulator of amino acid-derived gluconeogenesis [31]. Glutamine, alanine, lactate, and glycerol may be used for gluconeogenesis. The corresponding reserve pools of these substrates are restored during the feeding period and shaped by the dietary composition, then impacting on energy homeostasis over the fasting period. In the present study, when mice were fasted for 6 h, the circulating concentrations of the gluconeogenic substrate alanine were the lowest in liver GDH knockout mice under the PP diet. In these mice, amino acid-induced endogenous glucose production was impaired. However, alternative pathways could partially compensate, as revealed by liver metabolomics showing robust production of glucose-6-P. In response to fasting, insulin levels decreased only in mice under the PP diet while glycemia was maintained among groups; except for Hep-*Glud1*^*−/−*^ animals under the AP diet, which showed reduced blood glucose in response to fasting, although reaching levels of mice fed the PP regimen. Interestingly, this drop in blood glucose was associated with increased circulating ketone bodies, while fasting glycemia remained in the normal range. This reveals tight dependence on liver GDH during the fasting response, as confirmed by the impaired amino acid-induced endogenous glucose production in Hep-*Glud1*^*−/−*^ mice. Surprisingly, we did not observe compensatory increase of GDH activity in the kidney, the second most contributor to endogenous glucose production.

## 5 Conclusion

In conclusion, the study points to liver GDH for a swift adaptation to dietary protein source and ammonia handling. Plant-based diet is less effective in maintaining glycemia in the upper physiological range compared to an animal source of proteins, resulting in a fasting response rapidly engaging amino acid-derived gluconeogenesis dependent on hepatic GDH. This shows that, assuming a slight shift in the glycemia range, both diets can accommodate the need for systemic glucose supply, while functional GDH is required for safe ammonia disposal by the liver.

## CRediT authorship contribution statement

**Yan Zhou:** Writing – review & editing, Writing – original draft, Methodology, Investigation, Formal analysis, Data curation, Conceptualization. **Angela M. Ramos-Lobo:** Writing – review & editing, Writing – original draft, Methodology, Formal analysis, Conceptualization. **Tingting Fu:** Writing – original draft, Methodology, Formal analysis, Data curation. **Hector Gallart-Ayala:** Writing – original draft, Methodology, Formal analysis, Data curation. **Julijana Ivanisevic:** Writing – original draft, Supervision, Methodology, Formal analysis. **Pierre Maechler:** Writing – review & editing, Writing – original draft, Validation, Supervision, Resources, Project administration, Methodology, Funding acquisition, Formal analysis, Conceptualization.

## Ethics declaration

This study was conducted in accordance with the following guidelines for animal welfare and/or reporting: Swiss Federal Food Safety and Veterinary Office (FSVO). This study was approved by the Service de la consommation et des affaires vétérinaires (SCAV). (Approval No. GE/166/16).

## Funding

The work was funded by the 10.13039/100000001Swiss National Science Foundation (#310030_192486 to P.M.) and the canton of Geneva.

## Declarations

Authors state that.

• the work described has not been published previously except in the form of an abstract or an academic thesis.
• the article is not under consideration for publication elsewhere.
• the article's publication is approved by all authors and tacitly by the responsible authorities where the work was carried out.
• if accepted, the article will not be published elsewhere in the same form, in English or in any other language, including electronically, without the written consent of the copyright-holder.
• generative AI tools have not been used in the process of the manuscript preparation.

## Declaration of competing interest

The other authors declare that they have no conflicts of interest with the contents of this article.

## Data Availability

Data will be made available on request.
