# Supplementary material for: Transition to dietary plant-based proteins requires functional liver GDH for proper control of ammonia and energy homeostasis
Source: Mol Metab. 2026 Sep 4;113:102435. doi: 10.1016/j.molmet.2026.102435 (PMC13594857; doi:10.1016/j.molmet.2026.102435)

Zhou et al.

Supplemental Figures

Figure S1: Food intake recording over sequential dietary intervention periods. Time course of food intake by Control-*lox* and Hep-*Glud1*$^{-/-}$ mice fed alternatively for periods of 4 days with plant-based proteins (green), animal-based proteins (pink), and back to plant-based proteins. (**A**, **B**) Food consumption monitoring over the whole sequential dietary intervention expressed per hour (**A**) and per day (**B**). (**C**, **D**) Monitoring of food intake expressed in calories over the whole sequential dietary intervention expressed per hour (**C**) and per day (**D**). (**E**) Monitoring of hourly calorie intake zoomed in the AP period. (**F**) Monitoring of hourly calorie intake zoomed in the PP period. Values are mean ± SD, n=8.

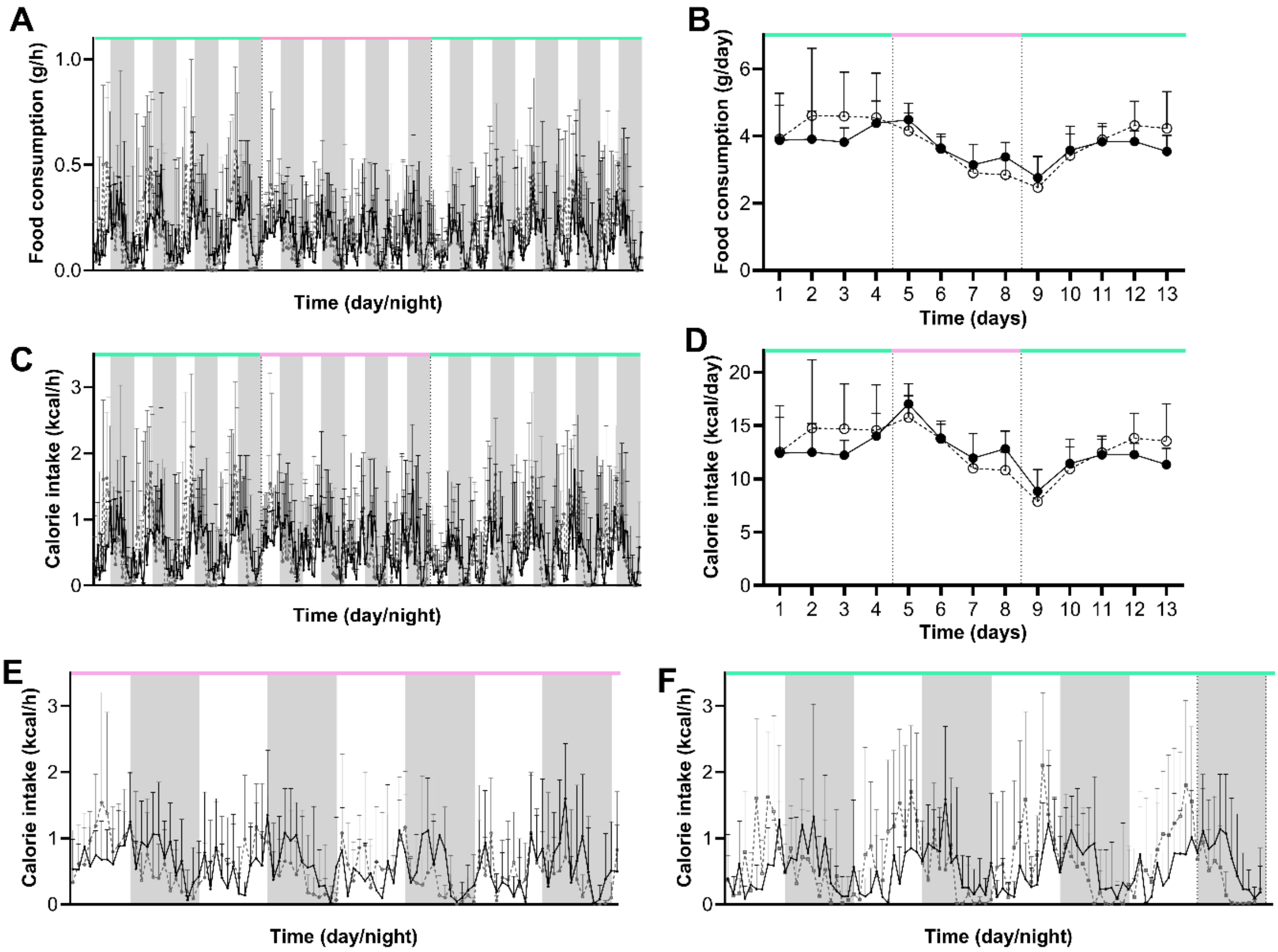

Figure S2: Changes in VCO_2 and physical activity over 24h in mice fed PP or AP diets. Metabolic and activity parameters of Control-*lox* and Hep-*Glud1*$^{-/-}$ mice fed either animal-based proteins (pink) or plant-based proteins (green) were recorded over 4 days and the last 24h period is shown with day and night quantifications. (**A**, **B**) VCO_2; (**C**, **D**) total movements; (**E**, **F**) Locomotor activity. Intervals between dash lines indicate the fasting period. AUC was calculated according to the Zeitgeber day and nighttime. Values are present as means ± SD, n=7-8. Two-way ANOVA was used for multiple comparisons of the genotypes of mice and diets. *p<0.05, **p<0.01, ***p<0.001 versus the corresponding Zeitgeber period; §p<0.05, §§p<0.01, §§§p<0.001 versus the corresponding genotype.

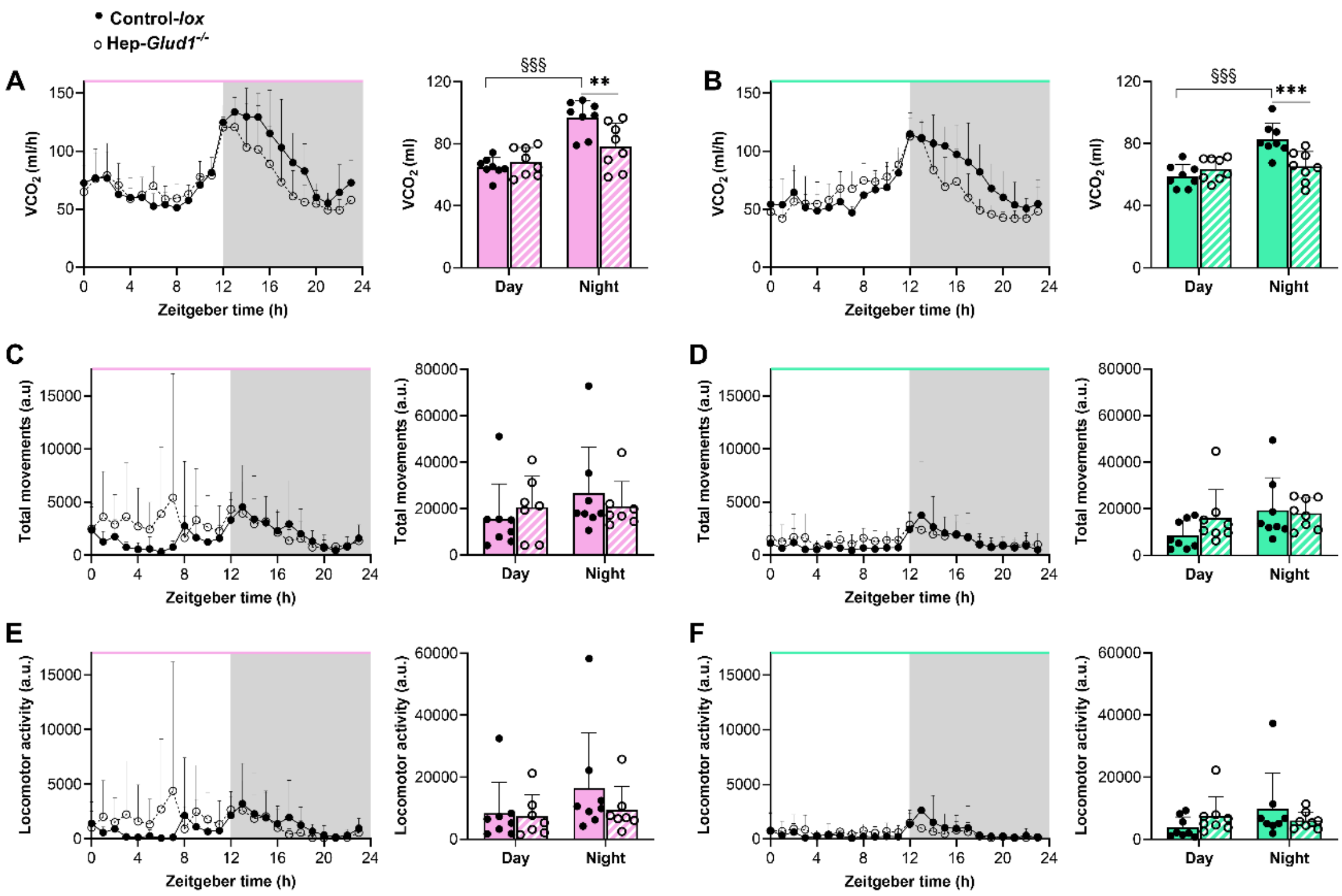

Figure S3: Liver and kidney protein levels assessed by immunoblotting. Control-*lox* and Hep-*Glud1*$^{-/-}$ mice mice fed diets composed of either animal-based proteins (AP) or plant-based proteins (PP). Tissues were collected for immunoblotting either in ad libitum fed conditions or after 6h of fasting. (**A**-**F**) Immunoblotting on liver extracts from fed mice for (**A**) glutamate dehydrogenase (GDH), (**B**) sirtuin SIRT4, (**C**) glucose-6-phosphatase (G6Pase), (**D**) phosphoenolpyruvate carboxykinase 1 (PCK1), (**E**) glutaminase 2 (GLS2), (**F**) glutamine synthetase (GS). (**G**-**L**) Immunoblotting on liver extracts from fasted mice for (**G**) GDH, (**H**) SIRT4, (**I**) G6Pase, (**J**) PCK1, (**K**) GLS2 and N-acetyl-glutamate synthase (NAGS), (**L**) GS, (**M**) Krüppel-like factor 15 (KLF15). (**N**-**P**) Immunoblotting on kidney extracts from fasted mice for (**N**) GDH, (**O**) G6Pase and PCK1, (**P**) GLS1 and GS.

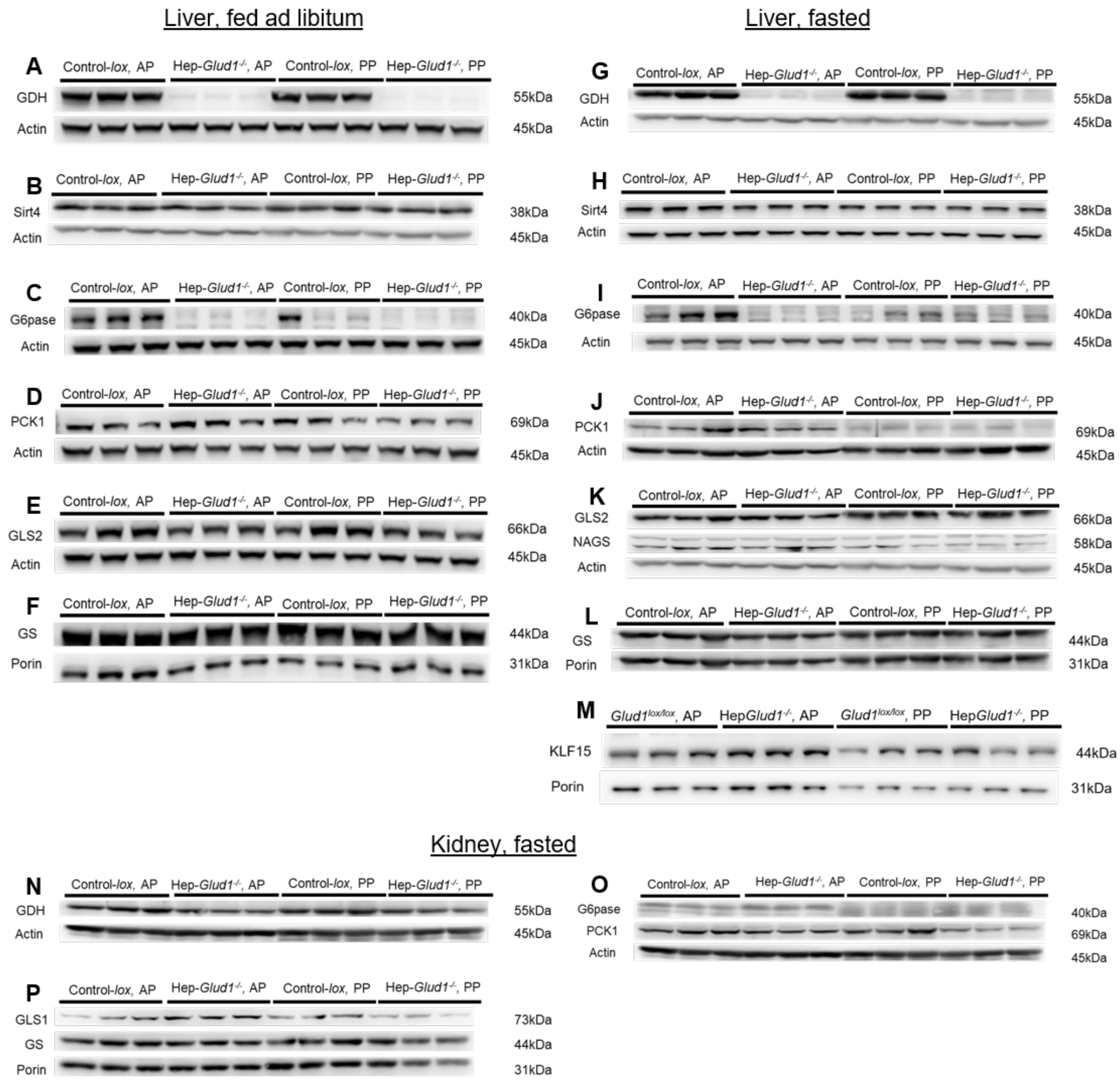

Figure S4: Quantification of liver and kidney protein levels assessed by immunoblotting. Control-*lox* and Hep-*Glud1*$^{-/-}$ mice mice fed diets composed of either animal-based proteins (AP, pink) or plant-based proteins (PP, green). Tissues were collected either in ad libitum fed conditions or after 6h of fasting. (**A**) N-acetyl-glutamate synthase (NAGS) in fasted conditions; liver (**B**) glucose-6-phosphatase (G6Pase), (**C**) phosphoenolpyruvate carboxykinase 1 (PCK1), (**D**) glutaminase 2 (GLS2), and (**E**) glutamine synthetase (GS) in fed conditions; kidney (**F**) GDH, (**G**) G6Pase, (**H**) PCK1, (**I**) glutaminase 1 (GLS1), (**J**) GS in fasted conditions. Values are means ± SD, n=3. Two-way ANOVA was used for Bonferroni and Sidak multiple comparisons tests of the genotypes of mice and dietary protein sources. *p<0.05, **p<0.01 versus the corresponding dietary protein sources; §p<0.05, §§p<0.01, §§§p<0.01versus the corresponding genotype.

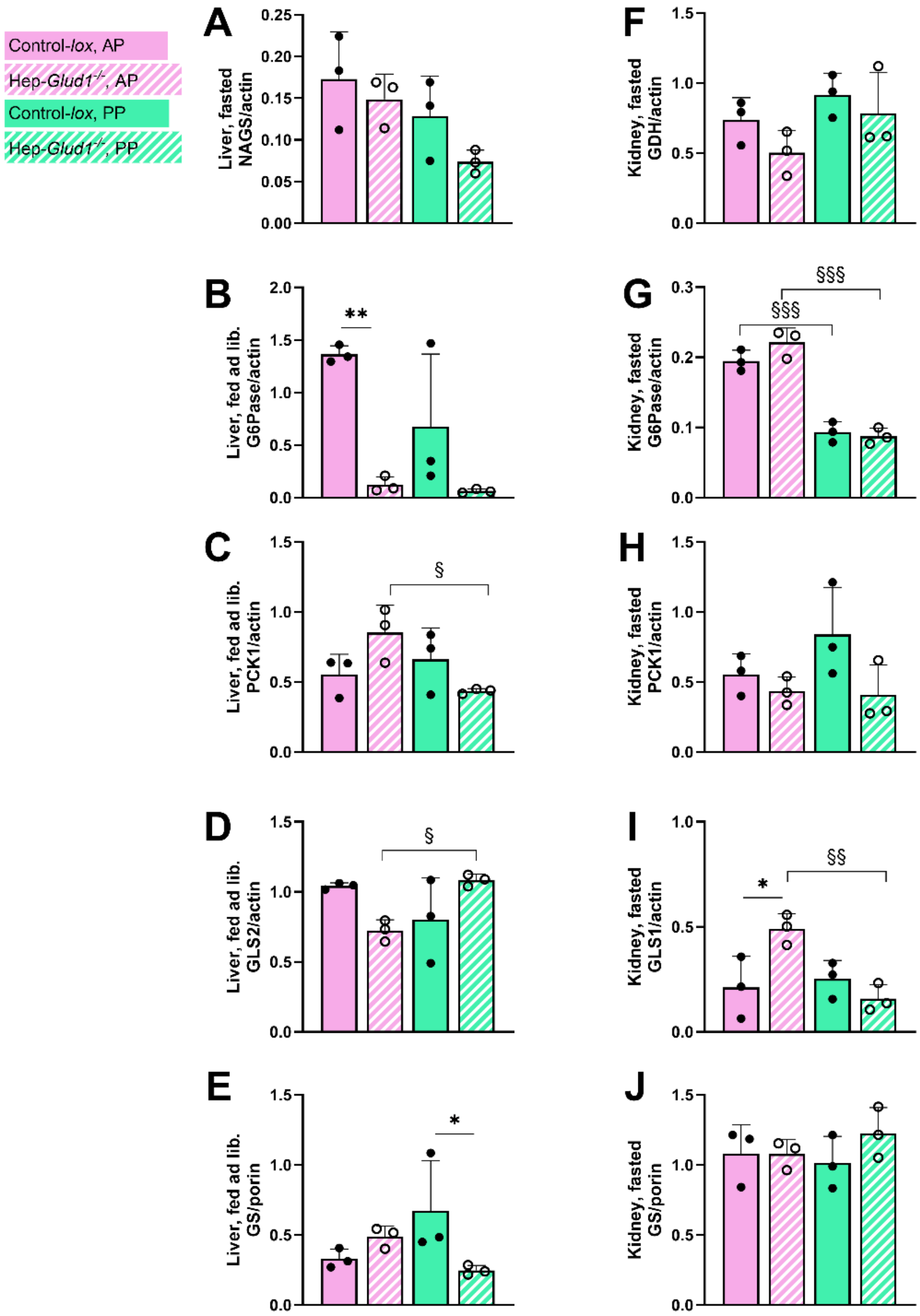

Figure S5: Effects of dietary protein source on liver metabolite levels. Control-*lox* and Hep-*Glud1*$^{-/}$ mice mice fed diets composed of either animal-based proteins (AP) or plant-based proteins (PP) were fasted for 6h before tissue collection and analyses. Liver metabolites were measured on liver extracts and are presented as schemes of biochemical pathways for glycolysis, the TCA cycle, and the ornithine cycle (see Supplemental Tables S2 and S3 for actual means ± SD values). Each circle shows the relative levels of metabolites in the liver of mice fed AP versus PP diets for (**A**) Control-*lox* mice and (**B**) Hep-*Glud1*$^{-/-}$ animals. Relative values range from -2.5 (blue) to +2.5 (red) and significant differences are indicated in the circle; n=5. Two-way ANOVA was used for Bonferroni and Sidak multiple comparisons tests of the genotypes of mice and dietary protein sources, §p<0.05, §§p<0.01.

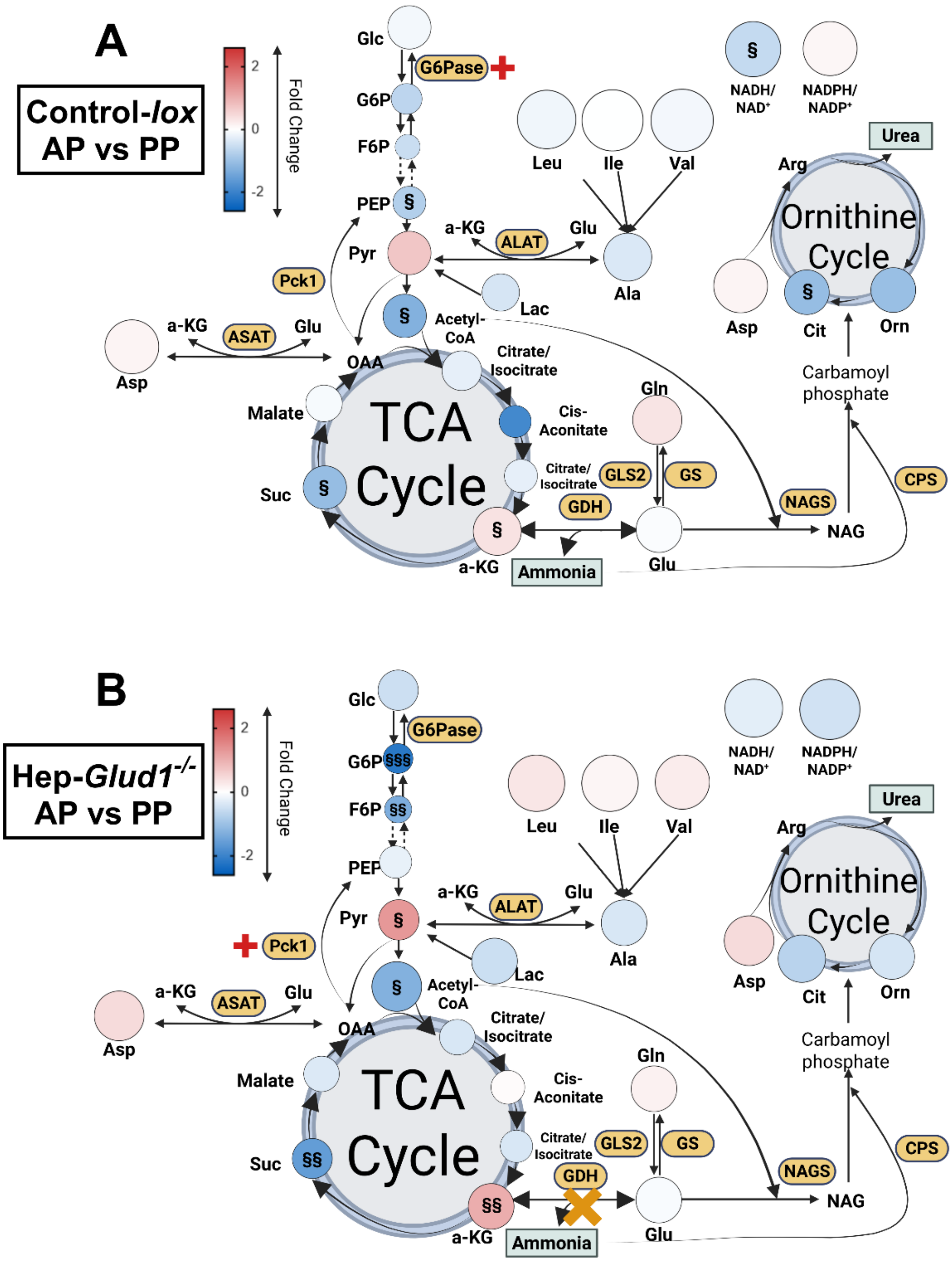

Figure S6: Representative images of sequential liver sections processed for immunostaining, GDH activity, and ToF-SIMS. Control-*lox* and Hep-*Glud1*$^{-/-}$ mice fed diets composed of either animal-based proteins (AP) or plant-based proteins (PP) were fasted for 6h before analyses: immunohistochemistry for GDH and GS (pericentral specific), *in situ* GDH enzymatic activity, ToF-SIMS imaging (regions of interest circled in white; scale bar = 100µm. (**A**, **B**) AP fed Control-*lox* analysed in periportal (A) and pericentral (B) regions. (**C**, **D**) AP fed Hep-*Glud1*$^{-/-}$ analysed in periportal (C) and pericentral (D) regions. ToF-SIMS color scale, 0 to maximum counts (mc).

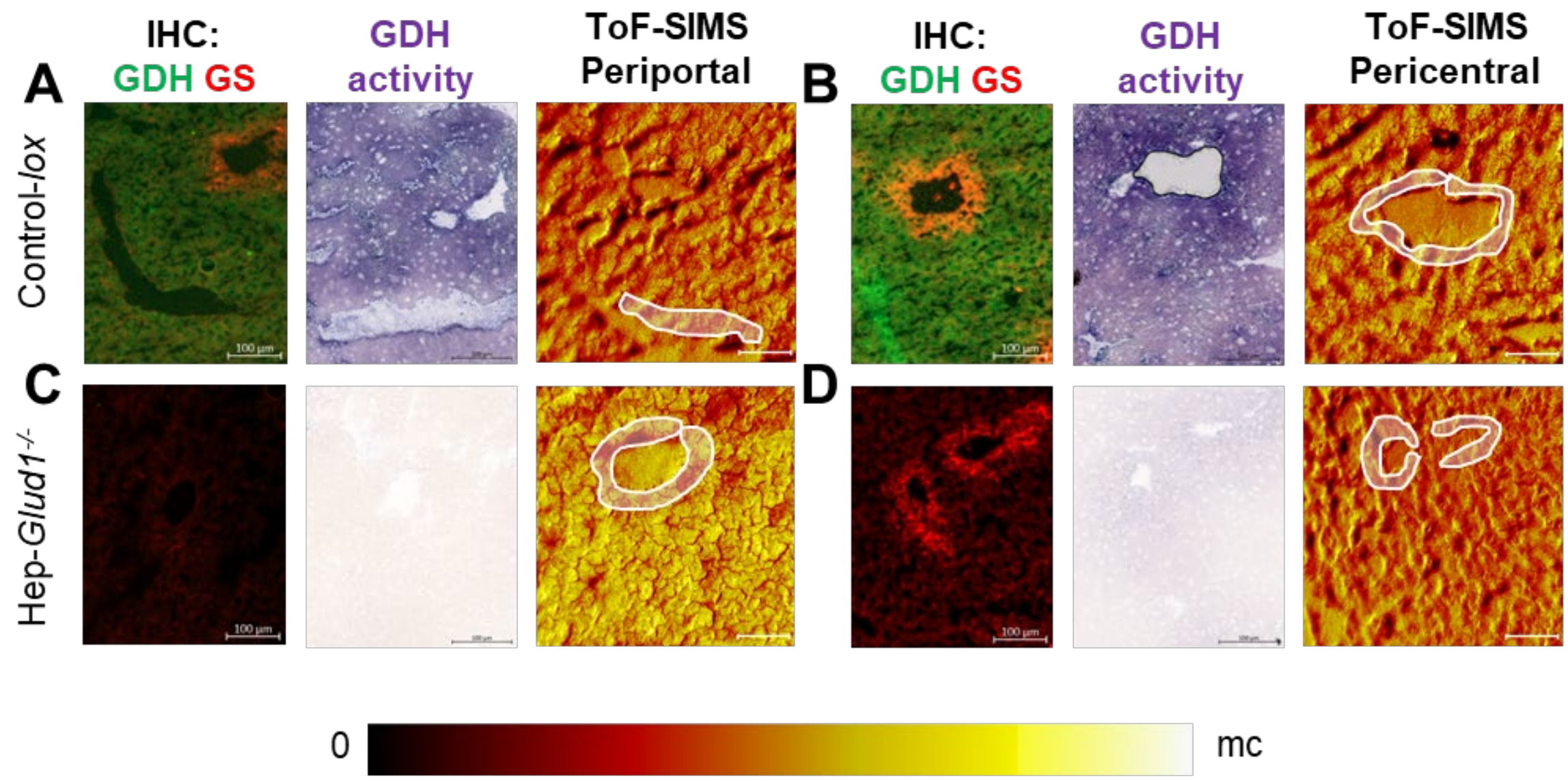

Supplementary Figure S7: Representative ToF-SIMS images of metabolite-specific fragments on liver periportal regions. Scale bar = 100μm; ToF-SIMS color scale, 0 to maximum counts (mc).

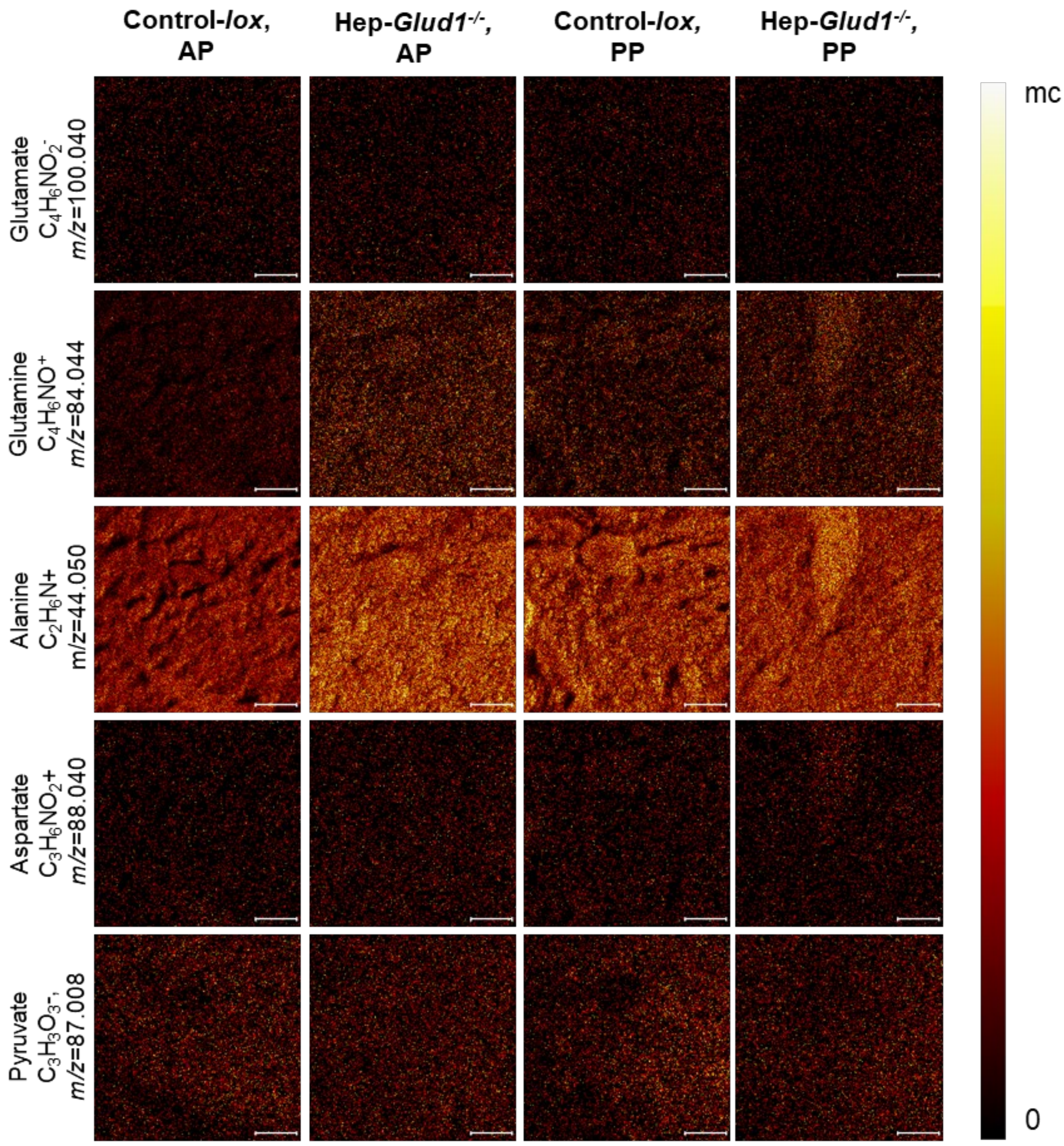

Supplementary Figure S8: Representative ToF-SIMS images of metabolite-specific fragments on liver pericentral regions. Scale bar = 100µm; ToF-SIMS color scale, 0 to maximum counts (mc).

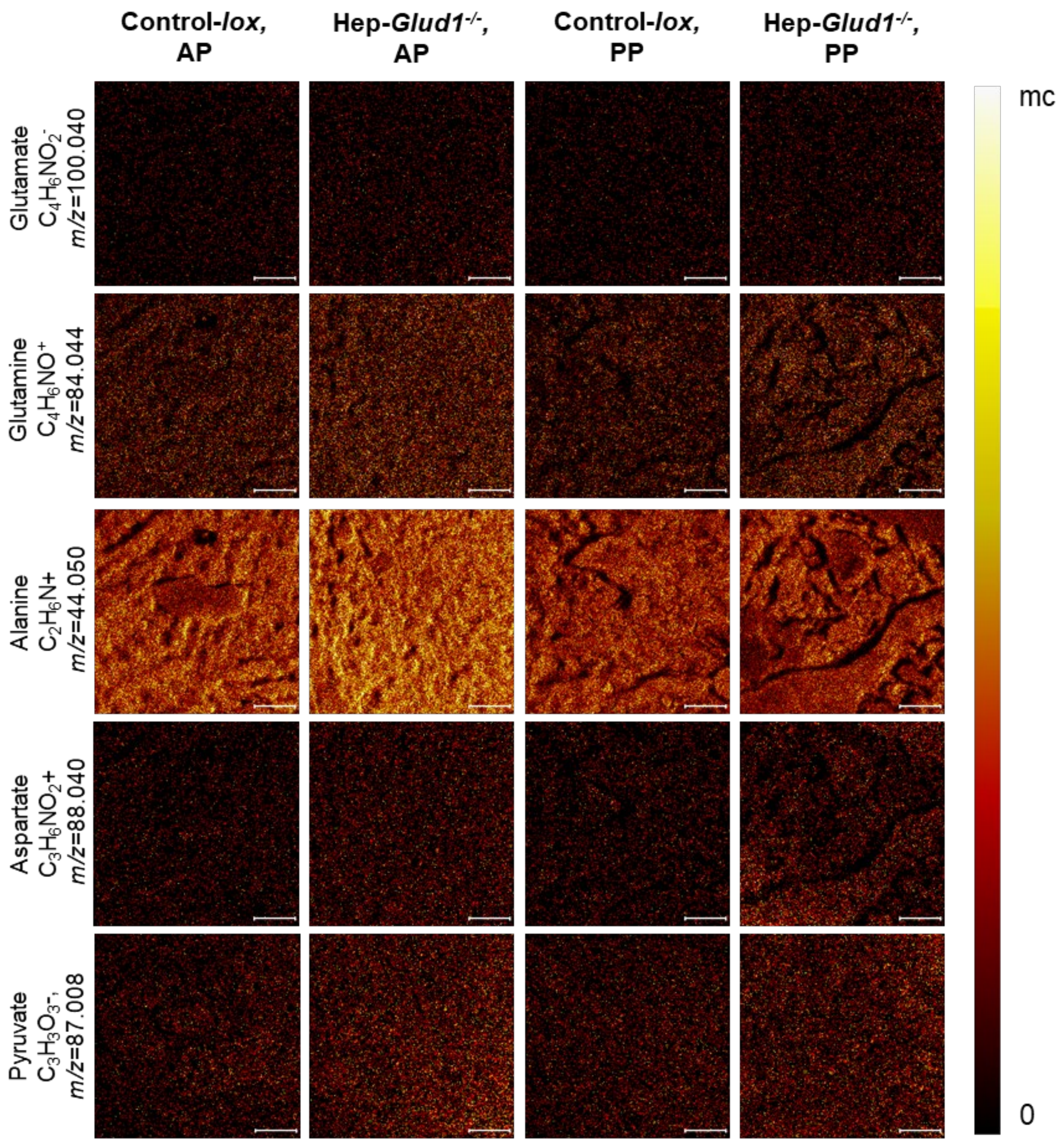

Figure S9: Selected peaks representing the liver metabolites of interest in fasted mice. ToF-SIMS spectrometry of (**A**) glutamate fragment ($C_4H_6NO_2^-$, *m/z*=100.040), (**B**) glutamine fragment ($C_4H_6NO_2^+$, *m/z*=84.044), (**C**) alanine fragment ($C_2H_6N^+$, *m/z*=44.050), (**D**) aspartate fragment ($C_3H_6NO_2^+$, *m/z*=88.040), (**E**) pyruvate fragment ($C_3H_3O_3^-$, *m/z*=87.008).

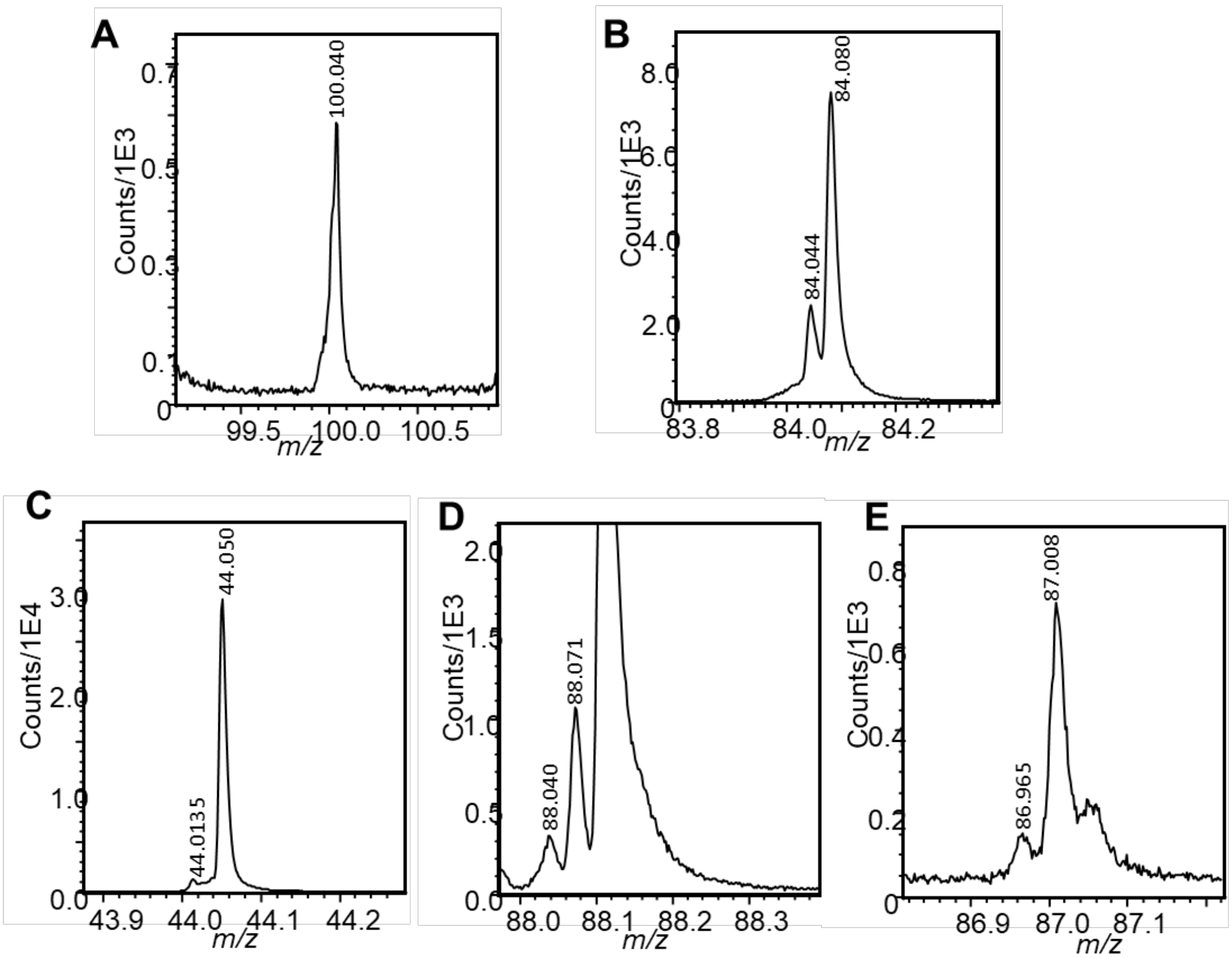

Supplement: Supplementary file 1 [file mmc1.pdf]
