# Supplementary material for: Transition to dietary plant-based proteins requires functional liver GDH for proper control of ammonia and energy homeostasis
Source: Mol Metab. 2026 Sep 4;113:102435. doi: 10.1016/j.molmet.2026.102435 (PMC13594857; doi:10.1016/j.molmet.2026.102435)
Supplement: Supplementary file 2 [file mmc2.pdf]

Zhou et al.

Supplemental Tables

Table S1: Overall centesimal composition of animal-based (D12450B) and plant-based (SAFE 150) diets, as provided by the supplier.

Nutrients	Research Diet D12450B	%	SAFE-diets SAFE 150	%
Carbohydrates	Sucrose	33.6	Cereals (mix)	72.7
Carbohydrates	Starch, Corn	29.9	*Sugars 3.4%*	
Carbohydrates	Lodex 10 (maltodextrin)	3.32	*Starch 41.1%*	
Fat	Lard	1.90	*Saturated 0.59%*	
Fat	Soybean Oil	2.37	*Unsaturated 3.45%*	
Fibers	Solka Floc, FCC200	4.74	*Fiber 4.1%*	
Proteins	Casein (lactic 30 Mesh)	19.0	Vegetal proteins	22.5
Proteins	L-cystine	0.3	*Crude 18.0%*	
Proteins			*Brewer yeast 1.14%*	
Proteins			Amino acids	0.5
Vitamins & Minerals	V10001C (vitamin mix)<br>S10026B (mineral mix)	0.095<br>4.74	Vitamins & Minerals	4.3
	Choline Bitartrate	0.19		
	Dye, Yellow FD&C #5	0.005		
		**100.0**		**100.0**

Supplementary Table S2: antibodies used for immunodetection on western blotting (WB) and immunohistochemistry (IHC).

**Target**	**Name**	**Supplier**	**Reference**	**Dilution**
Glutamate dehydrogenase (GDH)	Rabbit anti-GDH	Rockland	100-4158	1:2000 (WB)
Glutamate dehydrogenase (GDH)	Rabbit anti-GDH	Cell Signaling	D9F7P	1:500 (IHC)
Sirtuin 4 (Sirt4)	Goat anti-SIRT4	Abcam	ab10140	1:1000 (WB)
Glutaminase 2 (GLS2)	Rabbit anti-GLS2	Abcam	ab113509	1:2000 (WB)
Glutaminase 1 (GLS1)	Rabbit anti-GLS1-HRP	Abcam	ab200408	1:2500 (WB)
Glutamine synthase (GS)	Mouse anti-GS	Millipore	MAB302	1:1000 (WB)

Glutamine synthase (GS)	Mouse anti-GS	Millipore	MAB302	1:500 (IHC)
Glucose-6-phosphatase (G6pase)	Rabbit anti-G6Pase	University of Lyon	Dr. G. Mithieux	1:5000 (WB)
Phosphoenolpyruvate carboxykinase 1 (PCK1)	Rabbit anti-PCK1	Abcam	ab70358	1:2500 (WB)
N-acetyl-glutamate synthase (NAGS)	Rabbit anti-NAGS	Sigma	AV51183	1:4000 (WB)
Actin	Mouse anti-Actin	Millipore	MAB1501	1:5000 (WB)
Porin	Rabbit anti-Porin	Abcam	ab34726	1:2000 (WB)
Secondary	Anti-rabbit HRP	Sigma	A8275	1:4000 (WB)
Secondary	Anti-mouse HRP	Sigma	A5278	1:2500 (WB)
Secondary	Anti-goat HRP	Santa Cruz	sc-2056	1:3000 (WB)

Table S3: Peak assignments of the metabolite fragments or pseudo molecular species in positive and negative ToF-SIMS spectra.

Polarity	Metabolites	Formula	*m/z* (experimental, centroid)	*m/z* (theoretical)	Error (ppm)
Positive	Alanine	$C_2H_6N^+$	44.051	44.050	22.7
	Aspartate	$C_3H_6NO_2^+$	88.040	88.039	11.4
	Glutamine	$C_4H_6NO^+$	84.045	84.044	11.9
Negative	Pyruvate	$C_3H_3O_3^-$	87.010	87.008	23.0
	Glutamate	$C_4H_6NO_2^-$	100.039	100.040	10.0

Table S4: Amino acid contents of animal and plant-based diets (*estimate).

	Animal-based protein diet (g/100g diet)	Plant-based protein diet (g/100g diet)
Alanine	0.55	0.93
Arginine	0.65	0.98
Asparagine	0.76	0.5*
Aspartate	0.55	1.44
Cysteine	0.13	0.33
Glutamine	1.86	1.8*
Glutamate	2.26	3.53
Glycine	0.32	0.77
Histidine	0.49	0.43
Isoleucine	0.82	0.66
Leucine	1.71	1.38
Lysine	1.43	1.12
Methionine	0.55	0.38
Phenylalanine	0.91	0.78
Proline	1.92	1.33
Serine	1.08	0.81
Threonine	0.78	0.67
Tryptophan	0.23	2.00
Tyrosine	0.99	0.68
Valine	1.01	0.92
**Total**	**22.0**	**24.7**

Table S5: Liver glycolysis and TCA cycle metabolites measured by LC-MS performed on liver extracts. Control-*lox* and Hep-*Glud1*$^{-/-}$ mice mice under either animal-based proteins (AP) or plant-based proteins (PP) were fasted for 6h before tissue collection and analyses. Two-way ANOVA was used for multiple comparisons of the genotypes of mice and dietary protein sources. $^{*}p<0.05$, $^{**}p<0.01$ **versus the corresponding dietary protein sources**; $^{\S}p<0.05$, $^{\S\S}p<0.01$, $^{\S\S\S}p<0.001$ ***versus the corresponding genotype***; n=5.

	Concentration (µmol/g of proteins) or ratio for NAD(P)H/NAD(P)$^{+}$							
Metabolites	Control-*lox*, AP		Hep*Glud1*$^{-/-}$, AP		Control-*lox*, PP		Hep*Glud1*$^{-/-}$, PP	
	Mean	SD	Mean	SD	Mean	SD	Mean	SD
α-D-Glucose	231.88	66.66	209.67	111.42	253.25	15.31	301.77	12.38
Glucose-6-P	1.397	1.013	***1.392***§§§	0.767	**2.242****	0.985	***6.343*****,§§§	2.532
Fructose-6-P	1.903	1.135	***1.934***§§	1.291	**2.796***	0.992	***4.864****,§§	1.386
Phosphoenolpyruvate	***0.056***§	0.015	0.072	0.027	***0.097***§	0.027	0.085	0.015
Pyruvate	0.469	0.210	***0.496***§	0.184	0.285	0.095	***0.202***§	0.046
Acetyl-CoA	***0.036***§	0.013	***0.041***§	0.044	***0.091***§	0.033	***0.098***§	0.030
Citrate/Isocitrate	1.087	0.259	1.156	0.207	1.303	0.436	1.529	0.865
Cis-Aconitate	0.010	0.003	0.015	0.006	0.038	0.041	0.015	0.005
α-Ketoglutarate	**0.059***	0.007	***0.091****,§§	0.029	0.046	0.008	***0.044***§§	0.004
Succinate	***1.863***§	1.162	***1.502***§§	1.416	***3.912***§	0.872	***4.528***§§	1.241
Malate	4.668	0.619	5.060	1.847	4.982	1.188	6.488	2.484
Lactate	77.024	11.721	76.962	31.603	103.382	24.581	111.310	15.569
Glutarate	0.131	0.042	0.158	0.076	0.206	0.130	0.124	0.065
NAD^{+}	0.462	0.154	0.545	0.208	0.627	0.154	0.665	0.197
NADH	***0.195***§	0.086	0.269	0.225	***0.404***§	0.127	0.401	0.133
$NADH/NAD^{+}$	***0.410***§	0.027	0.440	0.088	***0.647***§	0.060	0.599	0.026
$NADP^{+}$	0.222	0.098	***0.253***§	0.055	0.248	0.021	***0.158***§	0.057
NADPH	0.399	0.150	0.618	0.323	0.413	0.090	0.534	0.135
$NADPH/NADP^{+}$	1.948	0.306	2.431	0.527	**1.660****	0.142	**3.555****	0.387

Table S6: Liver amino acids measured by LC-MS performed on liver extracts. Control-*lox* and Hep-*Glud1*$^{-/-}$ mice mice under either animal-based proteins (AP) or plant-based proteins (PP) were fasted for 6h before tissue collection and analyses. Two-way ANOVA was used for multiple comparisons of the genotypes of mice and dietary protein sources. Two-way ANOVA was used for multiple comparisons of the genotypes of mice and dietary protein sources. *p<0.05 **versus the corresponding dietary protein sources**; §p<0.05 ***versus the corresponding genotype***; n=5. Arginine levels could be detected only in 2 out of 5 Control-*lox* AP mice and in none of the Hep-*Glud1*$^{-/-}$ AP group (N.D., Not Detectable; N.A., Not Applicable).

	Concentration (µmol/g of proteins)							
Metabolites	Control-*lox*, AP		Hep-*Glud1*$^{-/-}$, AP		Control-*lox*, PP		Hep-*Glud1*$^{-/-}$, PP	
	Mean	SD	Mean	SD	Mean	SD	Mean	SD
Glutamine	23.673	6.720	22.977	4.617	18.770	5.588	20.436	4.804
Glutamate	5.375	0.990	6.140	3.082	5.596	0.510	6.464	5.264
Leucine	1.850	0.293	1.781	0.347	**2.057***	0.476	**1.435***	0.375
Isoleucine	0.968	0.170	0.853	0.230	0.976	0.231	0.785	0.234
Valine	1.693	0.332	1.727	0.184	1.855	0.476	1.466	0.345
Alanine	17.388	3.839	16.224	7.600	22.601	7.252	21.397	7.920
Aspartate	1.788	0.128	1.949	0.725	1.631	0.364	1.432	0.552
Citrulline	***0.157***§	0.072	0.137	0.031	***0.330***§	0.154	0.225	0.112
Arginine	0.023	N.A.	N.D.	N.A.	0.025	0.015	0.023	0.016
Ornithine	0.881	0.250	0.912	0.216	1.813	1.197	1.233	0.417
Asparagine	0.694	0.174	0.639	0.178	0.726	0.078	0.641	0.143
Phenylalanine	0.732	0.150	0.747	0.092	0.841	0.129	0.855	0.188
Tryptophan	0.290	0.081	0.270	0.035	0.257	0.031	0.294	0.049
Methionine	0.446	0.121	0.398	0.062	0.455	0.106	0.381	0.082
Taurine	130.21	32.18	119.89	16.04	125.15	24.96	158.66	25.91
Proline	***0.999***§	0.206	1.036	0.254	***1.607***§	0.482	1.130	0.411
Tyrosine	0.865	0.104	0.859	0.154	0.819	0.109	0.733	0.125
Threonine	1.284	0.255	1.291	0.242	1.469	0.236	1.553	0.248
Glycine	10.557	1.241	12.193	2.030	12.369	1.220	13.966	2.223
Serine	1.587	0.431	1.592	0.300	1.846	0.348	1.652	0.217
Lysine	2.502	0.571	2.785	0.683	**3.046***	1.329	**1.698***	0.235
Histidine	2.389	0.276	2.372	0.535	2.323	0.631	2.262	0.381
